# The effect of host factors on discriminatory performance of a transcriptomic signature of tuberculosis risk

**DOI:** 10.1016/j.ebiom.2022.103886

**Published:** 2022-02-18

**Authors:** Humphrey Mulenga, Andrew Fiore-Gartland, Simon C. Mendelsohn, Adam Penn-Nicholson, Stanley Kimbung Mbandi, Bhavesh Borate, Munyaradzi Musvosvi, Michèle Tameris, Gerhard Walzl, Kogieleum Naidoo, Gavin Churchyard, Thomas J. Scriba, Mark Hatherill, the CORTIS Study Team

**Affiliations:** a South African Tuberculosis Vaccine Initiative, Institute of Infectious Disease and Molecular Medicine, Division of Immunology, Department of Pathology, University of Cape Town, Anzio Road, Observatory, 7925, South Africa; b Vaccine and Infectious Disease Division, Fred Hutchinson Cancer Research Center, Fairview Ave. N., Seattle, WA 98109-1024, USA; c DST/NRF Centre of Excellence for Biomedical TB Research and SAMRC Centre for TB Research, Division of Molecular Biology and Human Genetics, Department of Biomedical Sciences, Faculty of Medicine and Health Sciences, Stellenbosch University, Francie Van Zijl Dr, Parow, 7505, South Africa; d Centre for the AIDS Programme of Research in South Africa (CAPRISA), Durban, Doris Duke Medical Research Institute, University of KwaZulu-Natal, 719 Umbilo Road, Durban 4001, South Africa; e MRC-CAPRISA HIV-TB Pathogenesis and Treatment Research Unit, Doris Duke Medical Research Institute, University of KwaZulu-Natal, 719 Umbilo Road, Durban 4001, South Africa; f The Aurum Institute, 29 Queens Rd, Parktown, Johannesburg, Gauteng 2194, South Africa; g School of Public Health, University of Witwatersrand, 27 St Andrews Road, Parktown, Johannesburg 2193, South Africa; h Department of Medicine, Vanderbilt University, Nashville, TN, USA

**Keywords:** *Mycobacterium tuberculosis*, Transcriptomic, Signature, RNA, Host factors, Performance

## Abstract

**Background:**

We aimed to understand host factors that affect discriminatory performance of a transcriptomic signature of tuberculosis risk (RISK11).

**Methods:**

HIV-negative adults aged 18–60 years were evaluated in a prospective study of RISK11 and surveilled for tuberculosis through 15 months. Generalised linear models and receiver-operating characteristic (ROC) regression were used to estimate effect of host factors on RISK11 score (%marginal effect) and on discriminatory performance for tuberculosis disease (area under the curve, AUC), respectively.

**Findings:**

Among 2923 participants including 74 prevalent and 56 incident tuberculosis cases, percentage marginal effects on RISK11 score were increased among those with prevalent tuberculosis (+18·90%, 95%CI 12·66−25·13), night sweats (+14·65%, 95%CI 5·39−23·91), incident tuberculosis (+7·29%, 95%CI 1·46−13·11), flu-like symptoms (+5·13%, 95%CI 1·58−8·68), and smoking history (+2·41%, 95%CI 0·89−3·93) than those without; and reduced in males (−6·68%, 95%CI −8·31−5·04) and with every unit increase in BMI (−0·13%, −95%CI −0·25−0·01). Adjustment for host factors affecting controls did not change RISK11 discriminatory performance. Cough was associated with 72·55% higher RISK11 score in prevalent tuberculosis cases. Stratification by cough improved diagnostic performance from AUC = 0·74 (95%CI 0·67−0·82) overall, to 0·97 (95%CI 0·90−1·00, *p* < 0·001) in cough-positive participants. Combining host factors with RISK11 improved prognostic performance, compared to RISK11 alone, (AUC = 0·76, 95%CI 0·69−0·83 versus 0·56, 95%CI 0·46−0·68, *p* < 0·001) over a 15-month predictive horizon.

**Interpretation:**

Several host factors affected RISK11 score, but only adjustment for cough affected diagnostic performance. Combining host factors with RISK11 should be considered to improve prognostic performance.

**Funding:**

Bill and Melinda Gates Foundation, South African Medical Research Council.

**Research in context**

**Evidence before this study**

We conducted a systematic review, by searching Medline, Scopus, Web of Science, and EBSCO with comprehensive search terms for “tuberculosis”, “diagnosis”, “prognosis”, “transcriptional”, “blood”, “signatures” and “performance”, for studies conducted in English and published between January 2005 and May 2019. Several host blood transcriptomic signatures with promising diagnostic and prognostic performance for tuberculosis disease have been developed. Non-TB pathogens, such as HIV and respiratory viruses, are thought to affect the readout of signatures like RISK11 that include interferon-stimulated genes. Only one previous study compared differential diagnostic accuracy among predefined population subgroups but did not evaluate the effect of several factors on the diagnostic performance of the signatures. Thus, a critical deficiency in transcriptomic biomarker development is the lack of understanding as to whether, and to what extent, host factors negatively affect discriminatory accuracy and contribute to a transcriptomic biomarker ‘performance ceiling’. It is not known whether tuberculosis signatures such as RISK11 should be adjusted for host factors that limit performance, or whether host factors that improve performance should be included in a combination clinical-transcriptomic signature to improve accuracy.

**Added value of this study**

To our knowledge, this is the first report that comprehensively evaluates the effect of host factors on discriminatory performance of transcriptomic signatures in HIV-negative individuals. We present evidence from a large longitudinal study in a tuberculosis-endemic setting that several tuberculosis-independent host factors affected the RISK11 signature readout in people without tuberculosis, but adjustment for these host factors did not alter discriminatory performance. By contrast, a tuberculosis-dependent host factor, cough, was associated with a 72·55% marginal increase in RISK11 score in prevalent tuberculosis cases and stratification by cough status showed significantly improved signature performance in people with cough. A combination signature including both host factors and RISK11, compared to RISK11 alone, did improve prognostic accuracy for incident tuberculosis that occurred at least 12 months after testing, a predictive horizon beyond which transcriptomic signature performance is known to deteriorate. However, the combination signature did not improve diagnostic or short-term prognostic accuracy of RISK11 within six months of tuberculosis disease, a time-frame within which transcriptomic signature performance is thought to be optimal.

**Implications of all the available evidence**

Although the host blood transcriptomic signature RISK11 is affected by tuberculosis-independent host factors, implementation as a triage or prognostic test for tuberculosis would not require adjustment for these host characteristics. By contrast, the tuberculosis-dependent host factor cough has a major impact on signature performance. Discriminatory performance in people with cough is excellent (AUC=0·97); poor performance (AUC=0·72) in people without cough suggests that RISK11 and similar transcriptomic signatures that detect interferon signalling genes may not be useful as triage tests for active case-finding of subclinical tuberculosis disease. This finding illustrates that cough status is a major contributor to the performance ceiling of host blood transcriptomic biomarkers of tuberculosis. Inclusion of host factors with the transcriptomic biomarker in a combination signature may mitigate the deterioration in signature performance over distant prognostic horizons, but does little to improve short-term performance. Future discovery and validation studies should examine the impact of host factors on performance of host blood transcriptomic signatures of tuberculosis across different populations and geographical settings.

Alt-text: Unlabelled box

## Introduction

Tuberculosis is a major public health problem, killing approximately 1·4 million people in 2019.^1^ Effective tuberculosis prevention and control requires rapid diagnosis and treatment of tuberculosis cases and early identification of individuals likely to develop tuberculosis so that they can be treated timeously to interrupt disease progression. However, quick and accurate tuberculosis diagnosis and prediction of progression to tuberculosis disease is hindered by inadequate available tests.^2^, ^3^, ^4^

Several studies have shown that host blood transcriptomic signatures can be used for both tuberculosis disease diagnosis and to predict progression to tuberculosis disease.^5^, ^6^, ^7^ However, tuberculosis-independent host factors may affect signature readout in individuals without tuberculosis (controls), whereas tuberculosis-dependent host factors would only affect signature readout in individuals with tuberculosis (cases), and potentially affect discriminatory performance. For example, factors independent of tuberculosis risk that increase biomarker scores among controls, such as viral infections,^8^ might increase the false-positive test rate, whilst host factors that decrease signature score in tuberculosis cases, such as increasing age and male sex,^9^^,^^10^ might reduce sensitivity. Tuberculosis-specific characteristics such as disease severity, might also influence the classification performance of the test.^11^ Therefore, host factors that shift biomarker distribution among cases and controls should be accounted for when evaluating discriminatory performance of diagnostic tests,^12^ to allow calculation of covariate-specific performance metrics (outcomes based on stratification of a specific covariate), or covariate-adjusted performance metrics (averaged outcomes which account for each covariate so that a better assessment of classification is obtained than the crude result).^13^ Host factors that contribute to accurate classification might be combined with the biomarker to create a combination risk score with improved diagnostic or predictive performance.^14^^,^^15^

We previously discovered and validated a 16-gene RNA host blood transcriptomic signature of risk that identified individuals with prevalent tuberculosis disease and predicted progression from *Mycobacterium tuberculosis* infection to tuberculosis disease.^16^ This 16-gene signature was adapted to real-time (RT) quantitative polymerase chain reaction (qPCR) platform and refined to an 11-gene (RISK11) signature with equivalent performance.^17^ RISK11 is comprised of interferon signalling pathway genes BATF2, ETV7, FCGR1C, GBP1, GBP2, GBP5, SCARF1, SERPING1, STAT1, TAP1, and TRAFD1 (Table S1). RISK11 was recently validated in a large multi-centre longitudinal study.^18^ We have previously shown that people living with HIV (PLHIV) have significantly higher RISK11 scores, compared to HIV-uninfected individuals;^19^ and male sex, older age, and lower HIV viral load are associated with reduced RISK11 score in PLHIV.^20^ RISK11 score may also be increased in the presence of upper respiratory viral pathogens.^8^ However, it is not known whether or which host factors affect RISK11 score; and the direction of associations is not known in HIV-negative individuals. Furthermore, the extent to which host factors affect discriminatory performance has not been quantified in HIV-uninfected populations. Specifically, it is not known whether RISK11 should be adjusted for host characteristics, or whether host characteristics should be included in a combination signature to improve performance.

If transcriptomic biomarkers are to be implemented as tuberculosis triage tests it would be important to account for confounding factors that affect signature performance. This study aimed to (i) identify and quantify the effect of tuberculosis-independent and tuberculosis-dependent host factors on RISK11 score in tuberculosis cases and healthy controls, (ii) quantify the effect of adjustment for host factors on diagnostic performance of RISK11 for prevalent and incident tuberculosis; and (iii) evaluate the effect on discriminatory performance of combining RISK11 with baseline risk factors for tuberculosis.

## Methods

### Ethics approval

This analysis is based on the dataset from a randomised clinical trial (CORTIS),^18^ conducted between September 2016 and December 2019 in South Africa, which evaluated the performance of RISK11 for diagnosis of prevalent tuberculosis and prediction of incident tuberculosis. The study was approved by Institutional Human Research Ethics Committees of the five participating sites and was also registered on ClinicalTrials.gov (NCT02735590). Written informed consent was sought and obtained from all participants.

### Study design and participants

The methodology and main results have been reported previously.^18^ In brief, HIV-uninfected adults between the ages of 18 and 60 years, with no history of tuberculosis disease within the last three years or other co-morbidities, were tested for RISK11 at baseline. RISK11 scores were measured by microfluidic RT-qPCR in whole blood RNA as previously described.^17^^,^^18^ Briefly, RISK11 is a model of multiple transcript pairs, each functioning as a “vote” for tuberculosis risk. The RISK11 score is the continuous proportion of positive transcript pair votes for tuberculosis risk, ranging from 0–100%. A score threshold can be set for the RISK11 assay to function as a qualitative (positive/negative) test for tuberculosis risk. All participants were screened for prevalent tuberculosis at baseline; those without prevalent tuberculosis were followed for up to 15 months for incident tuberculosis disease. Prevalent tuberculosis was defined as tuberculosis disease diagnosed within 30 days of enrolment (baseline); thereafter, any tuberculosis disease diagnosed was classified as incident disease. Controls were defined as participants without prevalent or incident tuberculosis including those with an unknown outcome at the end of study. Participants provided two expectorated sputum samples for tuberculosis investigation at baseline and end of study (Xpert MTB/RIF or Xpert MTB/RIF Ultra; Cepheid, Franklin Lakes, NJ); interim sputum investigation was symptom-triggered (liquid mycobacterial culture (MGIT, Becton-Dickinson, USA) and Xpert MTB/RIF or Xpert MTB/RIF Ultra). Participants presenting with any one or more symptoms of persistent unexplained cough, weight loss, chest pains, night sweats, fever, for two weeks or more; or any haemoptysis within the last two weeks, were defined as symptomatic. Flu-like symptoms other than those compatible with tuberculosis were also recorded. For this analysis, the microbiologically-confirmed tuberculosis disease endpoint was defined as one or more positive sputum samples by Xpert MTB/RIF, Xpert MTB/RIF Ultra, or MGIT culture. One-sample positive cases were tuberculosis cases in which collection of confirmatory sputum samples did not yield a confirmatory positive result within 30 days. Two-sample positive cases were tuberculosis cases in which collection of confirmatory sputum samples yielded a confirmatory positive result within 30 days. Note that the primary endpoint in the parent study (CORTIS) was two or more positive sputum samples and for this reason a sensitivity analysis was done for prediction of tuberculosis risk using baseline characteristics. A chest radiograph was performed in a sub-set of participants with microbiologically-confirmed tuberculosis and was interpreted by a clinical trial investigator. A positive chest radiograph was defined by any of the following features: hilar or paratracheal lymphadenopathy, miliary pattern, alveolar consolidation, cavitation, pleural effusion, apical shadows, Ghon focus, or calcified nodules.

### Statistical analysis

Statistical analyses were performed using STATA/IC version 16·1 (StataCorp., College Station, TX, USA) and MedCalc 20·023 (MedCalc Software Ltd, Ostend, Belgium). Descriptive statistics were computed as either mean and standard deviation (SD), or median and interquartile range (IQR), depending on the distribution, or as frequencies and percentages for categorical variables. The Wilcoxon Rank Sum and Kruskal Wallis tests were used to compare the distribution of RISK11 scores and other numerical variables between two and more than two groups, respectively. Categorical variables were compared using the Chi‐squared test, or Fisher's exact test when the expected frequencies were <5. Correlation between RISK11 score and continuous variables was measured using Spearman's rank correlation coefficient.

To quantify the associations between the dependent variable, RISK11 score and each of the predictor variables (host factors) in all participants and specific subgroups of interest (controls and prevalent and incident tuberculosis), univariable generalised linear models (GLMs) were employed (STATA *glm* command). A multivariable GLM was used to estimate the effect of baseline covariates on RISK11 score. Since RISK11 score is a continuous percentage ranging from 0–100%, which was scaled down to a proportion ranging between 0 and 1 for modelling purposes, the logit link function, binomial distribution family and a robust error term (vce-robust) were used in the models.^21^^,^^22^ The outcome measure was the percent marginal effect (increase/decrease) on RISK11 score associated with each predictor variable in the model (*margins* command). The model was built using the likelihood ratio test method. First, an initial model with just RISK11 was fitted. Next, nested models were fitted and compared to the initial model with likelihood ratios. The variable with the smallest additional Akaike Information Criterion (AIC) and biggest likelihood ratio, thus making the most significant contribution, was then added to the initial model. The process was repeated until no variable made a significant (*p* > 0·05) contribution to the previous model.

To adjust for covariates and evaluate the effect of covariates on the discriminatory performance of RISK11 for either prevalent or incident tuberculosis, ROC regression, using the *rocreg* command in STATA, was performed. The parametric option of the *rocreg* command was employed to allow adjustment for covariates and incorporation of sampling weights in the analysis. First, all variables significantly associated with RISK11 score among tuberculosis-negative controls and variables significantly associated with RISK11 among prevalent and incident tuberculosis cases in multivariable generalised linear regression were included in the ROC regression analyses. All these variables were included in both the “control population” (adjustment for control distribution) and “roc model” (covariates affecting ROC curve) parameters of the ROC regression analysis (Supplementary Table S7a). Next, all non-significant variables were removed from each respective section, one at a time until only significant variables remained. Significant variables in the control population as confirmed in ROC regression were included in covariate-adjusted ROC analyses. Variables significant (*p* < 0·05) in the roc model section of ROC regression analysis and therefore affecting the ROC curve and by extension discriminatory accuracy, were included in covariate-specific subgroup ROC analyses.^11^^,^^12^^,^^23^ Covariate- specific subgroup analyses were first performed at a 60% threshold level, which was the RISK11-positivity cut-off point, and thereafter performed at the optimal threshold levels for each subgroup to evaluate whether diagnostic performance improved with optimal covariate-specific thresholds compared to the original 60% thresholds. The Youden Index was used to compute RISK11’s optimal covariate-specific thresholds. Outcome measures for the ROC regression were the adjusted AUC and the host factors’ effect magnitude on the ROC curve.

To assess the use of baseline characteristics for the diagnosis and prediction of prevalent and incident tuberculosis, ROC analysis was performed. First, logistic and Cox proportional hazards regression models were constructed from covariates that were significant predictors of prevalent and incident tuberculosis (base models) respectively, using the likelihood ratio test method described above. A base model of risk for prevalent tuberculosis was constructed from age, BMI, and cough. A base model of risk for incident tuberculosis was constructed from BMI, smoking history, and previous tuberculosis history using the 15-month follow-up period; and the same base model was applied to the 6- and 12-month follow-up periods. Binary predictor variables included in the models had at least 10 tuberculosis events. The base models were then combined with RISK11 using logistic regression (incremental value method)^11^ and Cox regression for prevalent and incident tuberculosis respectively; in order to evaluate the improvement in classification performance. Thus, the outcome measure of interest was the AUC resulting from the combination risk score of RISK11 and the base model. A risk score (combination risk score or predicted risk of disease) was computed for each of the models that was used to construct the ROC curves. Optimal risk score threshold was based on the maximal Youden index. The area under the curve (AUC) for RISK11 alone and the base model alone were compared to that of the combined RISK11-base model AUC to assess the improvement in AUC. The AUCs were compared using the Delong et al method within MedCalc.^24^ The univariable and multivariable models as well as ROC analysis were adjusted with probability weights to reflect the CORTIS screening population.

Clarification is made here that covariate-adjustment in ROC analysis is different from using covariates in a predictive model or in incremental value analysis that also evaluate classification performance.^23^ When covariates are added in a model evaluating incremental value or prediction, such covariates contribute to the predicted probability of the outcome (combination risk score); usually computed with logistic regression. Thus, the resultant ROC curve for this combination score differs from a covariate-adjusted ROC curve of the test. Because covariates in a combination score contribute to classification, the combination score, may perform well even when the test is a poor classifier, provided that the covariate is a good classifier. In contrast, in covariate adjustment, the classification accuracy of the test is characterised conditional on the covariate.

Enrolment into CORTIS was dependant on RISK11 status and for purposes of conducting the study efficiently, roughly 79% of all eligible RISK11+ and only 13% of all eligible RISK11- participants were enrolled. Thus, the enrolled population was enriched with RISK11+ participants which required assignment of probability weights of 1·263 to RISK11+ and 7·920 to RISK11- individuals to obtain estimates of the screened population. A 0·05 significance level was used for statistical significance in all analyses. Unless otherwise stated, all analyses in this manuscript are based on the microbiologically-confirmed tuberculosis disease endpoint definition (≥1 positive sputum sample) to leverage the increased number of tuberculosis cases relative to the double-positive endpoint (≥2 positive sputum sample) used in the parent study (CORTIS). A sensitivity analysis was performed for baseline predictors of tuberculosis risk. Sample size calculation was performed to ensure that the Primary Objectives of the study could be addressed and did not consider the secondary analyses described here. For this reason, the third aim focused on testing whether baseline host characteristics could be used to improve performance and not necessarily to develop a validated model for prediction.

### Role of the funding source

The funders had no role in study design, data collection and analysis, decision to publish, or preparation of this manuscript.

## Results

### Baseline characteristics

20,207 volunteers were screened and 2,923 enrolled as previously described (Supplementary Figure S1). Prevalence of tuberculosis at baseline was 1·4% (74/2923, adjusted to reflect the screened population, see Methods) and cumulative tuberculosis incidence was 1·6% (56/2,849, adjusted) over 15 months. Participant baseline characteristics by tuberculosis status are shown in Table 1 (Supplementary Table S2). There were no significant baseline differences in ethnicity, tuberculosis contact history, and presence of flu-like symptoms among prevalent tuberculosis cases, those who progressed to incident tuberculosis, and controls without tuberculosis. Compared to controls, participants with prevalent tuberculosis and those who progressed to incident tuberculosis had lower BMI, higher RISK11 scores and majority were males. Additionally, compared to controls, participants with prevalent tuberculosis were older and had higher proportions of prior tuberculosis and symptoms; while those who progressed to incident tuberculosis had a higher proportion of smoking history.

**Table 1:** Baseline characteristics of enrolled participants by TB status.

Variable	Total	A) Prevalent TB	B) Incident TB	C) Control	A vs C	B vs C	A vs B vs C
	*n* = 2,923	*n* = 74	*n* = 56	*n* = 2,793	*P*-value	*P*-value	*P*-value
Age (median, IQR)	26 (22–33)	29 (24–36)	28 (22–37)	26 (22–33)	0·01	0·17	0·01
BMI (median, IQR)	23 (20–28)	21 (18–24)	20 (19–23)	23 (20–28)	<0·001	<0·001	<0·001
RISK11 Score (median, IQR)	26 (8–77)	87 (61–96)	67 (15–81)	24 (8–75)	<0·001	0·01	<0·001
Male sex (n, %)	1,338 (45·8)	47 (63·5)	33 (58·9)	1258 (45·1)	0·01	0·04	0·01
Ethnicity (n, %)							
Caucasian	4 (0·1)	0 (0)	0 (0)	4 (0·1)			
Mixed	968 (33·1)	34 (45·9)	26 (46·4)	908 (32·5)	0·08	0·18	0·06
Black	1,947 (66·6)	40 (54·1)	30 (53·6)	1,877 (67·2)			
Asian	4 (0·1)	0 (0)	0 (0)	4 (0·1)			
Smoking history (n, %)	1,478 (50·6)	45 (60·8)	41 (73·2)	1,392 (49·8)	0·08	0·01	<0·001
Prior TB (n, %)	230 (7·9)	19 (25·7)	8 (14·3)	203 (7·3)	<0·001	0·06	<0·001
TB contact history (n, %)	462 (15·8)	15 (20·3)	9 (16·1)	438 (15·7)	0·33	0·85	0·53
Flu-like symptoms (n, %)	134 (4·6)	4 (5·4)	1 (1·8)	129 (4·6)	0·78	0·52	0·66
***TB Symptoms***							
Chest pains (n, %)	30 (1·0)	4 (5·4)	0 (0)	26 (0·9)	0·01	1·00	0·01
Cough (n, %)	58 (2·0)	12 (16·2)	0 (0)	46 (1·6)	<0·001	1·00	<0·001
Fever (n, %)	3 (0·1)	1 (1·4)	0 (0)	2 (0·1)	0·08	1·00	0·13
Haemoptysis (n, %)	2 (0·1)	1 (0)	0 (0)	2 (0·1)	1·00	1·00	1·00
Loss of weight (n, %)	41 (1·4)	5 (6·8)	0 (0)	36 (1·3)	0·01	0·48	0·01
Night sweats (n, %)	32 (1·1)	7 (9·5)	1 (1·8)	24 (0·9)	<0·001	0·39	<0·001
Any symptom (n, %)	123 (4·2)	13 (17·6)	1 (1·8)	109 (3·9)	<0·001	0·72	<0·001

For continuous data, p values were computed using Wilcoxon Rank Sum test between two groups and Kruskal Wallis test for more than two groups. For categorical data, p values were computed using Fischer's exact test. *P*-values are not corrected for multiple comparisons. Participants that were not diagnosed with tuberculosis and did not complete follow-up for any reason were included in controls. Point estimates are computed using the enrolled population. See Supplementary Table 2 for adjusted point estimates to reflect screening population. IQR, inter-quartile range. BMI, body-mass index.

### Factors associated with RISK11 in all participants

**Figure 1:**
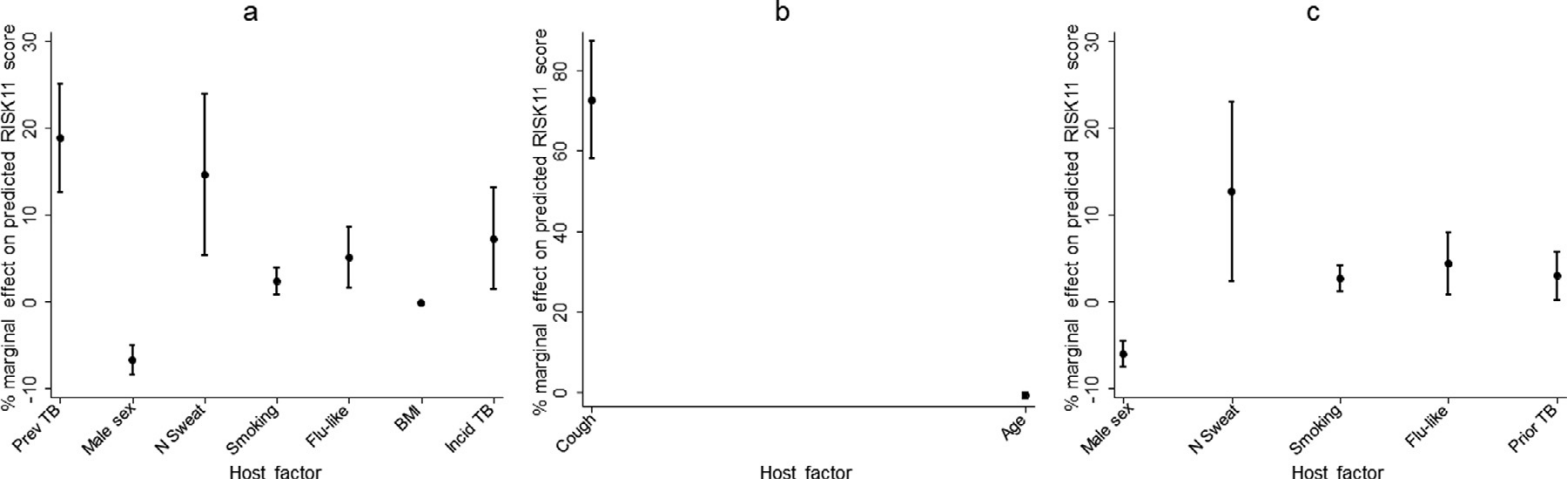
Predicted marginal effects on RISK11 by different host factors in (a) all participants, (b) participants with prevalent tuberculosis, and (c) participants without tuberculosis. Prev TB, Prevalent tuberculosis. N Sweat, Night sweats. Smoking, Smoking history. Flu-like, Flu-like symptoms. BMI, Body-mass index. Incid TB, Incident tuberculosis. Prior TB, Prior tuberculosis. The midline indicates the percentage marginal effect and the error bars indicate the 95% CIs.

First, the factors affecting RISK11 score in all participants, including tuberculosis cases and controls were assessed. In a multivariable generalised linear model, the percent marginal changes in RISK11 score were higher among participants with prevalent tuberculosis, those who progressed to incident tuberculosis, those with a smoking history, flu-like symptoms, or night sweats; and lower in males, and with every unit increase in BMI (Figure 1a; Multivariable GLM, *p* < 0·05). Age, ethnicity, prior tuberculosis disease, tuberculosis contact history, chest pains, cough, fever, haemoptysis, and subjective loss of weight were not independently associated with RISK11 score (Table 2).

**Table 2:** Univariable and multivariable generalised linear models of the predictors of RISK11 score in all participants.

Variable	n =2,923	Univariable Analysis		Multivariable Analysis		
		β. Coef. (95%CI)	*P*-value	β. Coef. (95%CI)	% Marginal effect (95%CI)	*P*-value
Age (median, IQR)	26 (22-33)	0·01 (0·001–0·01)	0·02	-		-
BMI (median, IQR)	23 (20-28)	-0·01 (-0·01–0·01)	0·56	-0·01 (-0·01–0·01)	-0·13 (-0·25–-0·001)	0·04
Male sex	1,338 (45·8)	-0·27 (-0·36–-0·19)	<0·001	-0·40 (-0·49–-0·30)	-06·68 (-8·31–-5·04)	<0·001
Race: Black (Reference)	1,947 (66·6)	-	-	-		-
Asian	4 (0·1)	-0·16 (-0·96–0·64)	0·69	-		-
Caucasian (n, %)	4 (0·1)	-0·49 (-1·34–0·35)	0·25	-		-
Mixed (n, %)	968 (33·1)	0·37 (0·29–0·46)	<0·001	-		-
Smoking history (n, %)	1,478 (50·6)	0·06 (-0·02–0·14)	0·16	0·14 (0·05–0·23)	2·41 (0·89–3·93)	<0·001
Prior TB (n, %)	230 (7·9)	0·24 (0·08–0·40)	0·01	-		-
TB contact history (n, %)	462 (15·8)	-0·06 (-0·16–0·05)	0·30	-		-
Flu-like symptoms (n, %)	134 (4·6)	0·32 (0·11–0·54)	0·01	0·30 (0·09–0·52)	5·13 (1·58–8·68)	0·01
Chest pains (n, %)	30 (1·0)	0·17 (-0·28–0·61)	0·46	-		-
Cough (n, %)	58 (2·0)	0·45 (0·13–0·78)	0·01	-		-
Fever (n, %)	3 (0·3)	0·89 (-0·29–2·07)	0·14	-		-
Haemoptysis (n, %)	2 (0·1)	-0·26 (-1·35–-1·16)	<0·001	-		-
Loss of weight (n, %)	41 (1·4)	0·1 (-0·25–0·45)	0·59	-		-
Night sweats (n, %)	32 (1·1)	0·92 (0·36–1·48)	0·01	0·87 (0·32–1·42)	14·65 (5·39–23·91)	0·01
Prevalent TB (n, %)	74 (2·5)	1·12 (0·75–1·49)	<0·001	1·12 (0·75–1·49)	18·90 (12·66–25·13)	<0·001
Incident TB (n, %)	56 (1·9)	0·41 (0·06–0·77)	0·02	0·43 (0·09–0·78)	7·29 (1·46–13·11)	0·01

IQR, inter-quartile range. BMI, body-mass index. β. Coef., Beta coefficient.

% Marginal effect. Percentage marginal change in RISK11 score associated with each respective predictor variable.

Next, the factors affecting RISK11 score in specific groups of interest were assessed, i.e., tuberculosis-dependant factors in prevalent and incident tuberculosis cases; and tuberculosis-independent factors in controls without tuberculosis.

### Factors associated with RISK11 in prevalent tuberculosis cases

Among participants with prevalent tuberculosis, RISK11 scores were significantly higher in symptomatic patients (13/74; median = 97·0%, IQR = 93·1–98·3%) compared to asymptomatic patients (median = 81·8%, IQR = 41·6–93·5%; Wilcoxon rank sum test, *p* < 0·001). No differences were observed in RISK11 scores in participants with or without a history of smoking, prior tuberculosis disease, or household tuberculosis contact history (Supplementary Table S3a). Of the 13 symptomatic patients, 10 were QuantiFERON-positive (QFT+). Median RISK11 scores were not different (Wilcoxon rank sum test, *p* = 0·80) between the 10 QFT+ (96·54%, IQR=82·68–100·00%) and the three QFT- (96·97%, IQR = 94·37–99·57%) patients.

Among the 28 prevalent tuberculosis cases in whom a chest radiograph was done, RISK11 scores were significantly higher in the 19 patients with a chest radiograph suggestive of tuberculosis (median = 97·8%, IQR = 68·4–99·6 vs median = 65·8, IQR = 15·6–85·3%; Wilcoxon rank sum test, *p* = 0·03, Supplementary Figure S2a). One-sample sputum positive cases had lower RISK11 scores (Supplementary Figure S3a), and a significantly lower proportion of chest radiographs suggestive of tuberculosis, compared to two-sample positive cases (33% vs 86%; Fisher's exact, *p* = 0·02).

In the analysis of factors affecting RISK11 score in the 74 prevalent tuberculosis cases, multivariable regression identified cough as the only factor affecting RISK11 score. Participants with a baseline cough were predicted to have a RISK11 score that was higher by 72·55% (95%CI 58·06–87·03) compared to those without cough (Figure 1b, Table 3a, Supplementary Table S4).

**Table 3:** Multivariable generalised linear models of the predictors of RISK11 score in prevalent TB cases and controls.

	Variable		Multivariable Analysis		
			β. Coef. (95%CI)	% Marginal effect (95%CI)	*P*-value
**A: Prevalent TB;** *n* = **74**	Age (median, IQR)	29 (24–36)	-0·03 (-0·06–0·01)	-0·65 (-1·34–0·04)	0·07
	Cough (n, %)	12 (16·2)	3·23 (2·45–3·94)	72·55 (56·08–87·03)	0·01
**B: Controls;** *n* = **2793**	Male sex	1,258 (45·0)	-0·36 (-0·45–-0·27)	-5·99 (-0·749–-4·50)	<0·001
	Smoking history (n, %)	1,392 (49·8)	0·16 (0·07–0·25)	2·74 (1·24–4·24)	<0·001
	Prior TB (n, %)	203 (7·3)	0·18 (0·01–0·35)	3·03 (0·25–5·82	0·03
	Flu-like symptoms (n, %)	129 (4·6)	0·26 (0·05–0·48)	4·39 (0·80–7·98)	0·02
	Night sweats (n, %)	24 (0·9)	0·76 (0·14–1·38)	12·69 (2·34–23·04)	0·02

Multivariable models for the factors associated with RISK11 score in (A) prevalent TB cases and (B) controls not diagnosed with either prevalent or incident TB. Complete univariable and multivariable models of this table are shown in Supplementary Tables 3 and 5.

IQR, inter-quartile range. BMI, body-mass index. β. Coef., beta coefficient.

% Marginal effect. Percentage marginal change in RISK11 score associated with each respective predictor variable.

### Factors associated with RISK11 in incident tuberculosis cases

Participants with incident tuberculosis were predominantly asymptomatic at baseline (Table 1) and showed no significant differences in baseline RISK11 score among those with or without a smoking history (median scores 66% vs 69%; Wilcoxon rank sum test, *p* = 0·39), prior tuberculosis disease (42% vs 70%; Wilcoxon rank sum test, *p* = 0·26), or a household tuberculosis contact history (66% vs 67%; Wilcoxon rank-sum test, *p* = 0·93, Supplementary Table S3b). A chest radiograph was performed at diagnosis in 36 participants and baseline RISK11 scores were not significantly different between the 25 patients with a positive chest radiograph and the 11 with a negative chest radiograph (median = 67·1%, IQR = 9·1–90·0% vs 48·9%, IQR = 15·2–72·7%; Wilcoxon rank sum test, *p* = 0·58; Supplementary Figure S2b). However, overall, one-sample sputum positive cases had lower RISK11 scores (Supplementary Figure S3e,f), and a significantly lower proportion of chest radiographs suggestive of tuberculosis, compared to two-sample positive cases (43% vs 86%; Wilcoxon rank sum test, *p* = 0·01). Stratification by diagnostic window showed that one-sample sputum positive incident cases diagnosed between months 2–6 and 7–12 (Supplementary Figure S3b and 3c, respectively) had lower RISK11 scores compared to two-sample positive cases but there was no difference in RISK11 score distribution between the one-sample and two-sample positive cases diagnosed between months 13–15 (Wilcoxon rank sum test, *p* = 0·89; Supplementary Figure S3d).

In the analysis of factors affecting RISK11 score in the 56 incident tuberculosis cases, flu-like symptoms and night sweats showed an association with RISK11 in univariable analysis (Supplementary Table S5). However, a multivariable model could not be fitted due to insufficient positive observations of participants with flu-like symptoms or night sweats *n* = 1).

### Tuberculosis-independent factors affecting RISK11 in controls

Among controls who remained tuberculosis-free through 15 months, those with any baseline symptom (109/2,793; median RISK11 scores of 48% vs 23%; Wilcoxon rank sum test, *p* < 0·001), baseline night sweats (71% vs 24%; Wilcoxon rank sum test, *p* = 0·01), flu-like symptoms (61% vs 23%; Wilcoxon rank sum test, *p* < 0·001), prior tuberculosis disease (43% vs 23%; Wilcoxon rank sum test, *p* = 0·02), and females (34% vs 16%; Wilcoxon rank sum test, *p* < 0·001) had significantly higher baseline RISK11 scores (Supplementary Table S3c). 2,603 of the 2,793 tuberculosis-free controls were asymptomatic and also free from flu-like symptoms compatible with tuberculosis, of which 960 (960/2,603) had elevated (≥60%) RISK11 scores (median = 84·0%, IQR = 70·1–94·4%).

Analysis of factors affecting RISK11 score in the 2,793 controls without tuberculosis using multivariable regression identified smoking history, prior tuberculosis, flu-like symptoms, night sweats, and sex as significant factors affecting RISK11 in controls. The percentage marginal effect on RISK11 score were higher in participants with a smoking history, prior tuberculosis, flu-like symptoms, or night sweats than in those without these characteristics; and lower in males than females (Multivariable GLM, *p* < 0·05; Figure 1c, Table 3b, Supplementary Table S6).

### Effect of host factors on RISK11 signature performance

Next, the effect of these baseline covariates on diagnostic and prognostic performance of RISK11 for tuberculosis was assessed using ROC regression analysis. RISK11 diagnostic performance (AUC 0·74, 95%CI 0·67–0·82) and prognostic performance through 15-months follow-up (AUC 0·56, 95%CI 0·46–0·68) for the one-sample positive tuberculosis cases was previously reported using nonparametric methods^18^. In the current analysis, a parametric method was used to allow adjustment for covariates and incorporation of sampling weights; the unadjusted parametric ROC analysis yielded results that were similar to the published non-parametric analysis with diagnostic AUC of 0·72 (95%CI 0·65–0·80) and prognostic AUC of 0·59 (95%CI 0·51–0·66).

**Figure 2:**
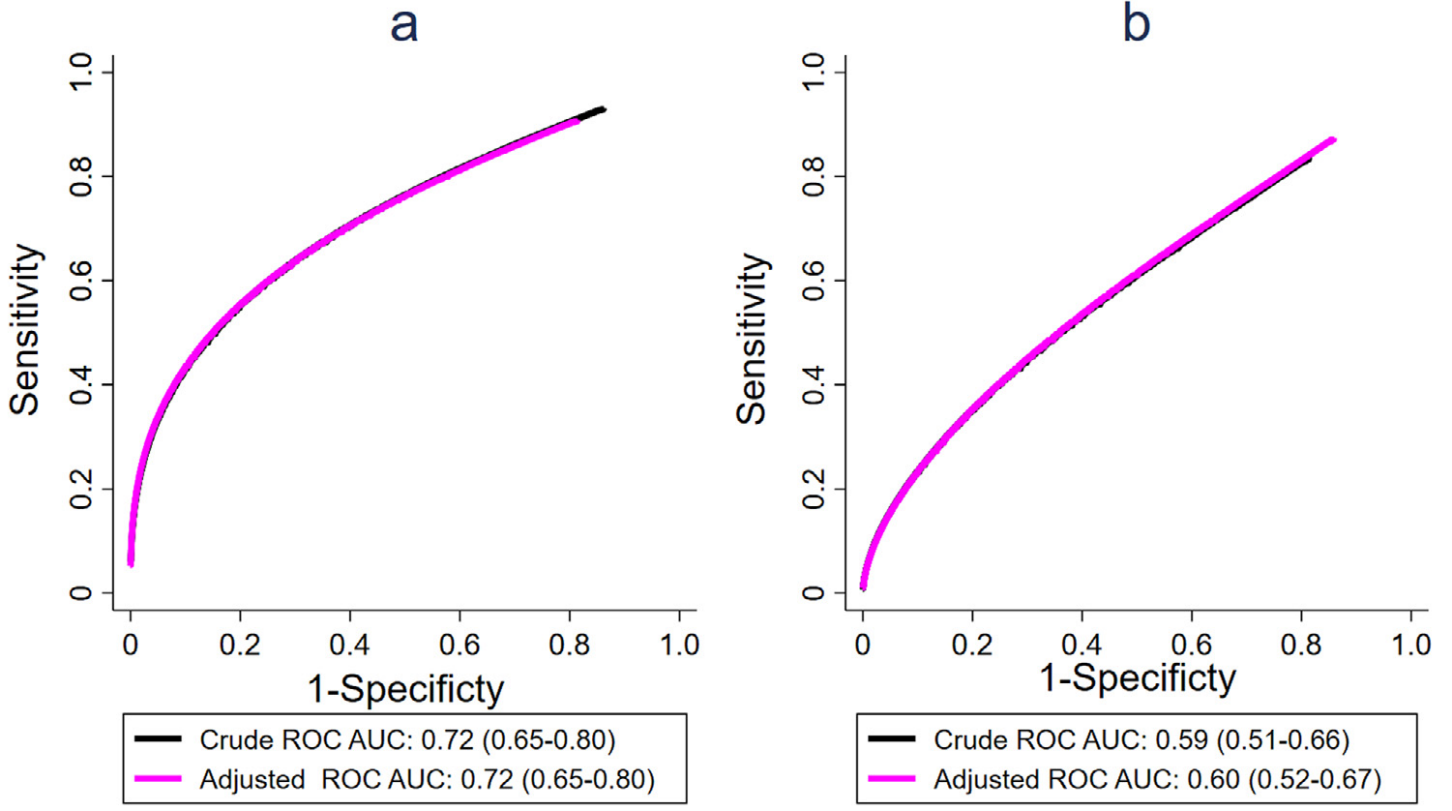
Crude and covariate-adjusted ROC curves for the discrimination of (a) prevalent TB from controls and (b) incident TB from controls. The crude and covariate-adjusted ROC curves are superimposed in both figures (a) and (b). The ROC curves are adjusted for BMI, sex, night sweats, haemoptysis, flu-like symptoms, and smoking history in both instances.

Adjustment for tuberculosis-independent host factors that significantly altered RISK11 distribution in controls (i.e. BMI, sex, night sweats, haemoptysis, flu-like symptoms, and smoking history) did not significantly alter the AUC for diagnostic performance for prevalent tuberculosis (AUC 0·72 vs 0·72;Delong method, *p* = 0·98). Similarly, the covariate-adjusted AUC for prognostic performance through 15-months follow-up (0·60, 95%CI: 0·52–0·67; Delong method, *p* = 0·94) did not significantly differ from the crude AUC (Figure 2).

**Figure 3:**
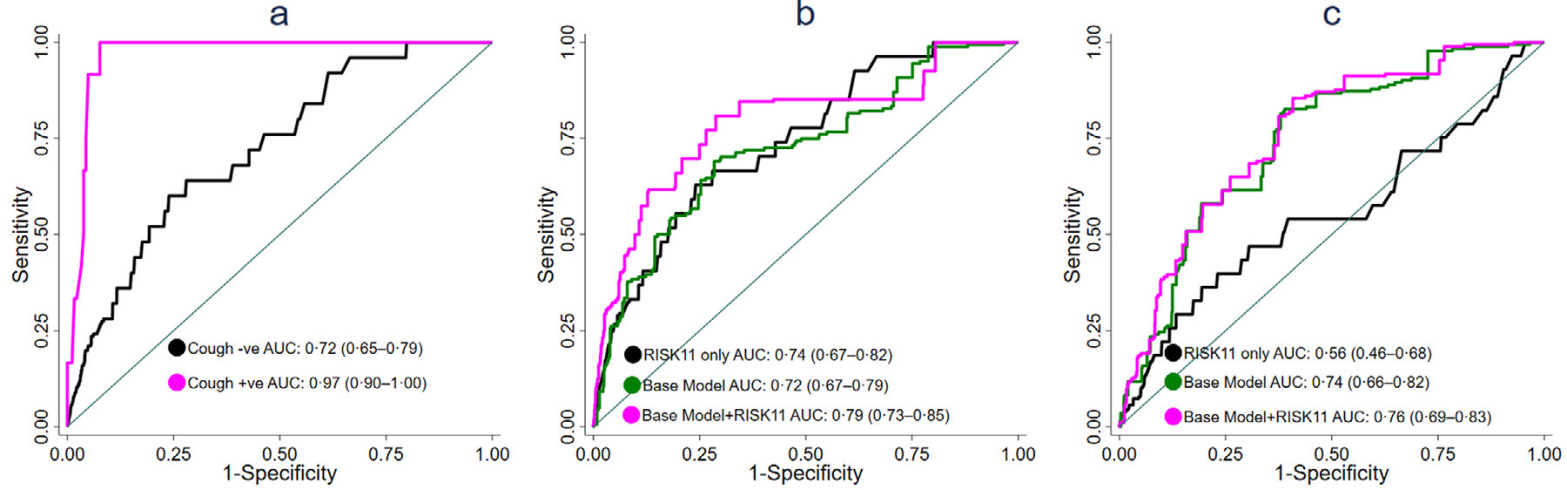
Performance of RISK11 when stratified by cough status and when combined with host factors. (a) Covariate-specific ROC curves for the diagnostic performance of RISK11 in cough-positive *n* = 58) and cough-negative (*n* = 2,865) individuals. Crude (RISK11 only), and combination ROC curves for discriminating (b) prevalent TB versus controls, and (c) incident TB versus controls through 15 months follow-up. Baseline model AUCs were derived from predictive models containing age, BMI, and cough for prevalent TB; and BMI smoking history, and previous TB history for incident TB.

Baseline cough and flu-like symptoms were the two factors that affected (Multivariable ROC regression, *p* < 0·001) discriminatory performance of RISK11 in prevalent and incident tuberculosis cases, respectively, in ROC regression (Supplementary Table S7a,b). Covariate-specific ROC curves computed for cough showed a high discrimination between cough-positive prevalent tuberculosis cases and cough-positive controls (AUC 0·97, 95%CI 0·90–1·00). In contrast, the AUC for discriminating cough-negative prevalent tuberculosis cases from cough-negative controls was 0·72 (95%CI 0·65–0·79; Delong method, *p* < 0·001, Figure 3a, Table 4c). Diagnostic accuracy improved from 62·2% in all to 94·6% in cough-positive individuals, at the 60% RISK11-positivity threshold (Table 4a vs 4c). The optimal cough-specific RISK11-positivity thresholds were 76% and 26% for cough-positive and cough-negative individuals respectively; and using these thresholds marginally improved covariate-specific performance of RISK11 (Table 4c vs 4d). Covariate-specific ROC curves were not computed for discriminating incident tuberculosis cases from controls in participants with and without flu-like symptoms, because only one individual with incident tuberculosis had flu-like symptoms**.**

**Table 4:** Crude and cough-specific performance estimates for RISK11 at various thresholds.

Statistic	a) Crude	b) Crude	c) Covariate-Specific		d) Covariate-Specific	
	RISK11(60)^†^*n* = 2,923	RISK11(26)^†^*n* = 2,923	Cough+ RISK11(60)^†^*n* = 58	Cough- RISK11(60)^†^*n* = 2,865	Cough+ RISK11(76)^†^*n* = *n* = 58	Cough- RISK11(26)^†^*n* = 2,865
**PR, (95%CI)**	4·8 (2·9–8·2)	4·86 (2·57–11·70)	10·7 (6·3–18·1)	3·9 (2·23–6·77)	13·4 (8·2–22·0)	4·4 (2·0–9·4)
**AUC, (95%CI)**	0·74 (0·67–0·82)		0·97 (0·90–100·0)	0·72 (0·65–0·79)	0·97 (0·90–100·0)	0·72 (0·65–0·79)
**Sensitivity, % (95%CI)**	33·2 (23·4–47·6)	62·9 (47·0–80·5)	100·0 (73·5–100·0)	28·1 (16·9–40·2)	100·0 (73·5–100·0)	60·0 (46·4–71·9)
**Specificity, % (95%CI)**	91·1 (91·0–91·2)	74·7 (73·1–76·4)	89·2 (76·4–96·4)	91·1 (90·0–92·1)	92·4 (79·2–97·6)	74·9 (73·3–76·5)
**PPV % (95%CI)**	4·9 (3·7–6·2)	3·3 (2·3–4·6)	37·5 (21·1–56·3)	4·0 (2·9–5·3)	46·2 (26·6–66·6)	3·0 (2·2–4·1)
**NPV % (95%CI)**	99·0 (98·5–99·4)	99·3 (98·9–99·7)	100 (86·8–100·0)	99·0 (98·4–99·4)	100 (89·1–100·0)	99·3 (98·7–99·7)
**Accuracy, % (95%CI)**	62·2 (60·4–64·0)	68·8 (67·1–70·5)	94·6 (85·6–98·9)	59·6 (57·8–61·4)	96·2 (88·1–99·6)	67·5 (65·8–69·2)

Covariate-specific subgroup analyses were first performed at 60% threshold level, which was the RISK11-positivity cut-off point, and thereafter performed at the optimal threshold levels for each subgroup, to evaluate whether diagnostic performance improved with optimal covariate-specific thresholds compared to the original 60% thresholds

†Numbers in brackets following RISK11 denote the RISK11-positivity threshold. Cough-specific thresholds in (d) were the optimal thresholds at the maximal Youden index.

PR, prevalence ratio. AUC, area under the curve. PPV, positive predictive value. NPV, negative predictive value.

### Combination of RISK11 with host factors

An assessment of whether combining RISK11 score with other baseline variables would improve discriminatory performance was made. Univariable and multivariable baseline predictors of either prevalent or incident tuberculosis and performance of these models is shown in Table 5. In multivariable analyses, the significant host predictors of prevalent tuberculosis were age, BMI, and cough, which formed the base model for prevalent tuberculosis (Table 5a). Combining the base model with RISK11 discriminated prevalent tuberculosis from controls with an AUC of 0·79 (95%CI 0·77–0·87), a non-significant (*p* = 0·06) increase of 5% compared to the 0·74 (95%CI 0·67–0·82) for RISK11 alone (Figure 3b).

**Table 5:** Performance of RISK11, base models, and combination models for TB disease.

Group	Model	Variable	Univariable AnalysisOR (95%CI) *P*-value		Multivariable Analysis aOR (95%CI) *P*-value		≥1 positive sputum sample)AUC (95%CI)	≥2 positive sputum sample)AUC (95%CI) †	P
**(a) Prevalent TB**	**RISK11 only**	RISK11	1·03 (1·02–1·04)	<0·001	–		0·74 (0·67–0·82)	0·77 (0·68–0·86)	0·54
	**Base Model**	Age	1·06 (1·03–1·08)	<0·001	1·06 (1·04–1·10)	<0·001	0·72 (0·65–0·79)	0·76 (0·69–0·84)	0·42
		BMI	0·91 (0·85–0·98)	0·01	0·90 (0·83–0·97)	0·01			
		Cough	5·01 (2·37–10·57)	<0·001	2·63 (1·10–6·27)	0·03			
	**Base Model + RISK11**	Age	1·06 (1·03–1·08)	<0·001	1·06 (1·03–1·09)	<0·001	0·79 (0·73–0·85)	0·83 (0·77–0·89)	0·38
		BMI	0·91 (0·85–0·98)	0·01	0·90 (0·84–0·97)	0·01			
		Cough	5·01 (2·37–10·57)	<0·001	2·23 (1·05–4·73)	0·04			
		RISK11	1·03 (1·02–1·04)	<0·001	1·03 (1·02–1·03)	<0·001			
**Group**	**Model**	**Variable**	**Univariable Analysis** **HR (95%CI)***P*-**value**		**Multivariable Analysis aHR/ (95%CI)***P*-**value**		**≥1 positive sputum sample)** **AUC (95%CI)**	**≥2 positive sputum sample)** **AUC (95%CI) †**	**P**
**(b) Incident TB through 6 months**	**RISK11 only**	RISK11	1·02 (1·00–1·04)	0·13	–		0·62 (0·45–0·79)	0·95 (0·92–1·00)	0·01
	**Base Model**	BMI	0·90 (0·77–1·05)	0·17	0·90 (0·80–1·02)	0·09	0·66 (0·49–0·83)	0·58 (0·34–0·82)	0·64
		Smoking	1·73 (0·34–8·73)	0·51	1·11 (0·29–4·18)	0·88			
		Prior TB	4·85 (0·71–33·12)	0·11	4·50 (0·71–28·47)	0·11			
	**Base Model + RISK11**	BMI	0·90 (0·77–1·05)	0·17	0·90 (0·80–1·02)	0·10	0·73 (0·57–0·89)	0·96 (0·85–1·00)	0·02
		Smoking	1·74 (0·34–8·80)	0·5	1·10 (0·29–4·19)	0·89			
		Prior TB	4·89 (0·7–33·87)	0·11	4·16 (0·64–26·91)	0·13			
		RISK11	1·02 (1·00–1·04)	0·13	1·02 (0·99–1·04)	0·17			
**(c) Incident TB through 12 months**	**RISK11 only**	RISK11	1·02 (1·00–1·03)	0·01	–		0·62 (0·51–0·73)	0·80 (0·65–0·94)	0·04
	**Base Model**	BMI	0·84 (0·76–0·93)	0·01	0·87 (0·80–0·94)	0·01	0·77 (0·67–0·87)	0·76 (0·63–0·89)	0·92
		Smoking	3·69 (1·21–11·26)	0·02	2·22 (0·76–6·49)	0·15			
		Prior TB	4·02 (1·26–12·84)	0·02	3·38 (1·01–11·31)	0·05			
	**Base Model + RISK11**	BMI	0·84 (0·76–0·93)	0·01	0·87 (0·80–0·94)	0·01	0·80 (0·70–0·90)	0·87 (0·76–0·98)	0·33
		Smoking	3·69 (1·21–11·26)	0·02	2·21 (0·76–6·44)	0·15			
		Prior TB	4·02 (1·26–12·84)	0·02	3·15(0·94–10·54)	0·06			
		RISK11	1·02 (1·00–1·03)	0·01	1·01 (1·00–1·03)	0·02			
**(d) Incident TB through 15 months**	**RISK11 only**	RISK11	1·01 (1·00–1·02)	0·01	–		0·56 (0·46–0·68)	0·63 (0·47–0·80)	0·30
	**Base Model**	BMI	0·87 (0·80–0·94)	0·01	0·90 (0·83–0·97)	0·01	0·74(0·66–0·82)	0·80 (0·70–0·90)	0·34
		Smoking	3·92 (1·76–8·74)	0·01	2·62 (1·13–6·08)	0·03			
		Prior TB	3·09 (1·27–7·51)	0·01	2·61 (1·03–6·57)	0·04			
	**Base Model + RISK11**	BMI	0·87 (0·80–0·94)	0·01	0·90 (0·82–0·97)	0·01	0·76 (0·69–0·83)	0·82 (0·73–0·92)	0·32
		Smoking	3·92 (1·76–9·08)	0·01	2·61 (1·14–6·24)	0·03			
		Prior TB	3·09 (1·27–7·51)	0·01	2·46 (1·00–6·20)	0·05			
		RISK11	1·01 (1·00–1·02)	0·01	1·01 (1·00–1·02)	0·02			

All modelling data shown in this table is based on the one-sample positive endpoint definition (≥1 positive sputum sample) which was the primary endpoint for this analysis. Point estimates for prevalent tuberculosis are adjusted odds ratios (aOR) and those for incident (Tables b–d) tuberculosis are adjusted hazard ratios (aHR). AUCs shown with ‘†’ are based on a sensitivity analysis computed using the two-sample positive endpoint definition (≥2 positive sputum samples) which was the primary endpoint in CORTIS. The corresponding model output data for the two-sample positive endpoint are not shown. BMI, body-mass index. AUC, area under the curve. P values comparing the AUCs for the one-sample versus two-sample positive endpoint definition were computed using the Delong method in MedCalc.

Similarly, host predictors included in the incident tuberculosis base model were BMI, smoking history, and previous tuberculosis history (Table 5b–d). Combining the incident tuberculosis base model with RISK11 significantly improved discrimination between incident tuberculosis and controls, from AUCs of 0·62 (95%CI 0·51–0·73) and 0·56 (95%CI 0·46–0·68) for RISK11 alone, to AUCs of 0·80 (95%CI 0·70–0·90; Delong method, *p* = 0·02) and 0·76 (95%CI 0·69–0·83; Delong method, *p* < 0·001) for the combination model, over 12- and 15-month prognostic horizons respectively (Table 5c,d, Figure 3c). However, combination of the incident tuberculosis base model with RISK11 did not significantly improve prediction compared to RISK11 alone (Delong method, *p* = 0·11) through a 6-month follow-up period (Table 5b).

## Discussion

We have shown that although several host factors affected RISK11 readout, adjustment for tuberculosis-independent host factors affecting controls did not change diagnostic or prognostic performance of the RISK11 transcriptomic signature. However, stratification for cough status, a tuberculosis-dependent factor that was associated with a 72·55% marginal increase in RISK11 score in those with prevalent tuberculosis, significantly improved discriminatory accuracy in individuals with cough. However, diagnostic performance in individuals without cough was poor.

We also showed that although certain host factors affecting RISK11 score are also associated with tuberculosis risk, incorporation of these host factors into a combination signature did not significantly improve diagnostic performance for prevalent tuberculosis. By contrast, combining baseline host factors with RISK11 significantly improved discrimination of incident tuberculosis from controls compared to RISK11 alone over the longer 12- and 15-month predictive horizons. These findings may also be generalisable to other transcriptomic signatures that, like RISK11, include interferon-stimulated genes.

These findings build upon our previous work that showed the effect of HIV infection^19^ and upper respiratory viral pathogens on RISK11 score;^8^ and on the work of others who have evaluated the effect of host factors on performance of transcriptomic signatures^25^ and combined biomarkers and clinical variables to improve prediction of tuberculosis risk and treatment outcomes.^15^^,^^26^^,^^27^ In addition to identifying host characteristics associated with changes in RISK11 score and tuberculosis risk, we have quantified the effect of these host factors on the ability of RISK11 to discriminate between participants with prevalent tuberculosis or incident tuberculosis from controls without tuberculosis.

Viral or other infections in participants without tuberculosis cannot be excluded as the cause of the raised RISK11 scores since we did not test for respiratory or other pathogens. In a sub-study that co-enrolled 286 participants and tested for upper respiratory tract pathobionts in nasopharyngeal and oropharyngeal swabs, RISK11 was able to differentiate between participants with prevalent tuberculosis and those with no pathobionts detected or only bacterial pathobionts. However, RISK11 could not differentiate between participants with prevalent tuberculosis and those with upper respiratory tract viruses.^8^ Similarly, HIV infection has been associated with raised RISK11 scores, especially in those with uncontrolled viral load, and was associated with diminished performance in one study where most participants were not on antiretroviral therapy,^19^ but associated with good performance in another study where most participants were on stable antiretroviral therapy.^20^

In a recent study, it was shown that transcriptomic signatures have good discriminatory capacity for tuberculosis disease in participants presenting with symptoms compatible with tuberculosis.^28^ We also previously showed that diagnostic performance of RISK11 for prevalent tuberculosis was superior in symptomatic tuberculosis cases, compared to cases of subclinical tuberculosis,^18^ which form a large proportion of tuberculosis cases in community prevalence surveys.^29^ Here, we evaluated which symptom component underlies this difference in performance. Cough was the only host factor that was significantly associated with a raised RISK11 score in participants with prevalent tuberculosis, with a 72·55% marginal increase in RISK11 score compared to cough-negative cases, and cough affected discriminatory performance in ROC regression. Cough is the most common manifesting symptom of symptomatic tuberculosis disease and thus typically distinguishes symptomatic from subclinical presentation, which may be associated with less severe disease, as suggested by higher Xpert/MTB RIF Ct values in subclinical disease.^30^ The superior performance of RISK11 in participants with symptomatic tuberculosis disease which may be associated with severe inflammation, suggests induction of interferon signalling resulting in elevated signature scores that drive the superior discriminatory performance. We found that RISK11 had excellent diagnostic performance at a 76% RISK11-positivity threshold in a small number of cough-positive individuals and similar performance at 26% RISK11-positivity threshold in cough-negative individuals to that of the crude estimates at a 26% RISK11-positivity threshold (Table 4). Although discriminatory performance for screening of asymptomatic individuals might be improved by using a different threshold than for symptomatic patients, the use of multiple thresholds for different populations would complicate interpretation and likely hinder implementation in the field.

Several host characteristics, including some factors associated with tuberculosis risk, were associated with a significantly increased or decreased RISK11 score in controls. Although we surmised it might be important to incorporate covariate information in assessing discriminatory performance, we showed that RISK11 performance was not different between covariate-adjusted and crude ROC curves (Figure 2).

Several studies have shown that combining biomarkers with host risk factors may significantly improve classification capacity.^14^^,^^15^^,^^26^^,^^27^^,^^31^ Sivakumaran *et al* found that signature performance for predicting tuberculosis treatment outcomes was improved when they combined host-derived biomarkers with patient characteristics.^27^ We demonstrated that combining the significant clinical predictors of incident tuberculosis through 12- and 15-months with RISK11 significantly improved discriminatory capacity compared to RISK11 alone, but not for incident tuberculosis through 6-months, or for prevalent tuberculosis. This important finding demonstrates that a classification model consisting of RISK11 plus baseline characteristics may improve discriminatory capacity for predicting incident tuberculosis through longer predictive horizons at which transcriptomic signature performance deteriorates. Alternatively, a signature discovered in asymptomatic participants might be required in a classification model to improve short term classification of incident tuberculosis.^32^

Weaknesses of our study may include the fact that we used a tuberculosis disease endpoint definition based on one positive sputum sample, as used in the public health system. However, one-sample sputum positive cases were predominantly subclinical, with fewer chest radiographs suggestive of tuberculosis, and lower RISK11 scores compared to the two-sample sputum positive cases (Supplementary Figure S3). It is therefore not surprising that the one-sample-positive endpoint showed poorer RISK11 performance compared to a two-sample-positive endpoint used in the CORTIS trial, which increases the potential for host factors to improve performance in a combination model. Furthermore, although we found that RISK11 performance was better in participants with a cough, this was based on a relatively small sample size. Baseline predictors performed well relative to RISK11 for prediction of tuberculosis risk over longer time-frames. It should be noted that this study reports the training cohort for these host factors and validated performance would require testing in an independent cohort. Strengths of this study include the large study sample, large number of tuberculosis cases, and the fact that the study recruited from five geographically distinct areas throughout South Africa with unique population demographics. These findings from five geographically distinct sites are broadly representative of community settings with high prevalence of undiagnosed subclinical tuberculosis in South Africa. They may not be applicable to other countries with low rates of prevalent and incident tuberculosis.

This study highlights that the discriminatory performance of RISK11 and potentially other transcriptomic signatures may be affected by host factors and the tuberculosis endpoint definition. Although this study showed that only cough influenced discriminatory performance of RISK11, further work may be warranted in high-risk populations, for example PLHIV or other co-morbidities such as diabetes mellitus. Future transcriptomic signature discovery studies should not ignore host characteristics in their design. Evidence from this study suggests that presence or absence of cough has a major impact on diagnostic performance for tuberculosis disease, which might severely limit the utility of transcriptomic biomarkers for triage and active case-finding approaches for subclinical tuberculosis, which forms a large proportion of prevalent tuberculosis in endemic communities.^29^

## Contributors

MH and TJS conceived and directed the study. MT, GW, KN, and GC were responsible for site-level activities, including recruitment, clinical management, and data collection. HM, SCM, SKM, and MM provided operational or laboratory support and project management. AFG provided statistical support. HM analysed the data and wrote the first draft of the manuscript. HM, AFG, SCM, APN, SKM, BB, MM, MT, GW, KN, GC, TJS and MH had full access to the data, and reviewed, revised, and approved the manuscript before submission.

## Declaration of interests

AP-N, GW, GC, TJS, and MH report grants from the Bill & Melinda Gates Foundation, during the conduct of the study; AP-N and GW report grants from the South African Medical Research Council, during the conduct of the study; GW and TJS report grants from the South African National Research Foundation, during the conduct of the study. In addition, AP-N and TJS have patents of the RISK11 and RISK6 signatures pending; GW has a patent “TB diagnostic markers” (PCT/IB2013/054377) issued and a patent “Method for diagnosing TB” (PCT/IB2017/052142) pending. All other authors had nothing to disclose.

## Appendix Supplementary materials

Image, application 1

## References

[1] STATA (2015). https://www.stata.com/manuals14/rfracreg.pdf.

[2] Hamada Y., Cirillo D.M., Matteelli A., Penn-Nicholson A., Rangaka M.X., Ruhwald M. (2021). Tests for tuberculosis infection: landscape analysis. Eur Respir J.

[3] Treatment-Action-Group (2019).

[4] Nema V. (2012). Tuberculosis diagnostics: challenges and opportunities. Lung India.

[5] Mulenga H., Zauchenberger C.Z., Bunyasi E.W. (2020). Performance of diagnostic and predictive host blood transcriptomic signatures for tuberculosis disease: a systematic review and meta-analysis. PLoS One.

[6] Gupta R.K., Turner C.T., Venturini C. (2020). Concise whole blood transcriptional signatures for incipient tuberculosis: a systematic review and patient-level pooled meta-analysis. Lancet Respir Med.

[7] Warsinske H., Vashisht R., Khatri P. (2019). Host-response-based gene signatures for tuberculosis diagnosis: a systematic comparison of 16 signatures. PLoS Med.

[8] Mulenga H., Musvosvi M., Mendelsohn S.C. (2021). Longitudinal dynamics of a blood transcriptomic signature of tuberculosis. Am J Respir Crit Care Med.

[9] Molony R.D., Nguyen J.T., Kong Y., Montgomery R.R., Shaw A.C., Iwasaki A. (2017). Aging impairs both primary and secondary RIG-I signaling for interferon induction in human monocytes. Sci Signal.

[10] Klein S.L., Flanagan K.L. (2016). Sex differences in immune responses. Nat Rev Immunol.

[11] Janes H., Longton G., Pepe M. (2009). Accommodating covariates in receiver operating characteristic analysis. Stata J.

[12] Janes H., Pepe MS. (2009). Adjusting for covariate effects on classification accuracy using the covariate-adjusted receiver operating characteristic curve. Biometrika.

[13] Pardo-Fernandez J.C., Rodríguez-Álvarez M., Keilegom I. (2014). A review on ROC curves in the presence of covariates. Revstat Statist J.

[14] Patel V.B., Singh R., Connolly C. (2010). Comparison of a clinical prediction rule and a LAM antigen-detection assay for the rapid diagnosis of TBM in a high HIV prevalence setting. PLoS One.

[15] Tenforde M.W., Gupte N., Dowdy D.W. (2015). C-reactive protein (CRP), interferon gamma-inducible protein 10 (IP-10), and lipopolysaccharide (LPS) are associated with risk of tuberculosis after initiation of antiretroviral therapy in resource-limited settings. PLoS One.

[16] Zak D.E., Penn-Nicholson A., Scriba T.J. (2016). A blood RNA signature for tuberculosis disease risk: a prospective cohort study. Lancet.

[17] Darboe F., Mbandi S.K., Thompson E.G. (2018). Diagnostic performance of an optimized transcriptomic signature of risk of tuberculosis in cryopreserved peripheral blood mononuclear cells. Tuberculosis.

[18] Scriba T.J., Fiore-Gartland A., Penn-Nicholson A. (2021). Biomarker-guided tuberculosis preventive therapy (CORTIS): a randomised controlled trial. Lancet Infect Dis.

[19] Darboe F., Mbandi S.K., Naidoo K. (2019). Detection of tuberculosis recurrence, diagnosis and treatment response by a blood transcriptomic risk signature in HIV-infected persons on antiretroviral therapy. Front Microbiol.

[20] Mendelsohn S.C., Fiore-Gartland A., Penn-Nicholson A. (2021). Validation of a host blood transcriptomic biomarker for pulmonary tuberculosis in people living with HIV: a prospective diagnostic and prognostic accuracy study. Lancet Glob Health.

[21] Baum CF. (2008). Stata tip 63: modeling proportions. Stata J.

[22] Chen K., Cheng Y., Berkout O., Lindhiem O. (2017). Analyzing proportion scores as outcomes for prevention trials: a statistical primer. Prev Sci.

[23] Janes H., Pepe M.S. (2008). Adjusting for covariates in studies of diagnostic, screening, or prognostic markers: an old concept in a new setting. Am J Epidemiol.

[24] DeLong E.R., DeLong D.M., Clarke-Pearson DL. (1988). Comparing the areas under two or more correlated receiver operating characteristic curves: a nonparametric approach. Biometrics.

[25] Turner C.T., Gupta R.K., Tsaliki E. (2020). Blood transcriptional biomarkers for active pulmonary tuberculosis in a high-burden setting: a prospective, observational, diagnostic accuracy study. Lancet Respir Med.

[26] Gupta R.K., Calderwood C.J., Yavlinsky A. (2020). Discovery and validation of a personalized risk predictor for incident tuberculosis in low transmission settings. Nat Med.

[27] Sivakumaran D., Jenum S., Vaz M. (2020). Combining host-derived biomarkers with patient characteristics improves signature performance in predicting tuberculosis treatment outcomes. Commun Biol.

[28] Sutherland J.S., van der Spuy G., Gindeh A. (2021). Diagnostic accuracy of the Cepheid 3-gene host response fingerstick blood test in a prospective, multi-site study: interim results. Clin Infect Dis.

[29] Van der W.M., Moyo S. (2020).

[30] Du Bruyn E., Ruzive S., Lindestam Arlehamn C.S. (2021). Mycobacterium tuberculosis-specific CD4 T cells expressing CD153 inversely associate with bacterial load and disease severity in human tuberculosis. Mucosal Immunol.

[31] Bedell R.A., van Lettow M., Meaney C. (2018). Predictive value of C-reactive protein for tuberculosis, bloodstream infection or death among HIV-infected individuals with chronic, non-specific symptoms and negative sputum smear microscopy. Tropical medicine & international health. TMIH.

[32] Kwan P.K.W., Periaswamy B., De Sessions P.F. (2020). A blood RNA transcript signature for TB exposure in household contacts. BMC Infect Dis.

