# Supplementary material for: The effect of host factors on discriminatory performance of a transcriptomic signature of tuberculosis risk
Source: eBioMedicine. 2022 Feb 18;77:103886. doi: 10.1016/j.ebiom.2022.103886 (PMC8861653; doi:10.1016/j.ebiom.2022.103886)
Supplement: Supplementary file 1 [file mmc1.pdf]

THE EFFECT OF HOST FACTORS ON DISCRIMINATORY PERFORMANCE OF A TRANSCRIPTOMIC SIGNATURE OF TUBERCULOSIS RISK

Humphrey Mulenga, MPH[1], Andrew Fiore-Gartland, PhD[2], Simon C. Mendelsohn, MBChB[1], Adam Penn-Nicholson, PhD[1], Stanley Kimbung Mbandi, PhD[1], Bhavesh Borate, MBBS[2], Munyaradzi Musvosvi, PhD[1], Michèle Tameris, MBChB[1], Gerhard Walzl, PhD[4], Kogieleum Naidoo, PhD[5,6], Gavin Churchyard, MBBCh[3,7,8], Thomas J. Scriba, PhD[1], Mark Hatherill, MD[1], and the CORTIS Study Team.

ONLINE DATA SUPPLEMENT

Contents

The CORTIS Study Team 2

Supplementary Tables 5

- Table S1: The 11 transcripts comprising the RISK11 signature of TB risk and their putative functions 5
- Table S2: Baseline characteristics of enrolled participants by TB status adjusted to reflect screening population. 6
- Table S3: Comparison of distribution of RISK11 scores by baseline characteristics in (a) Prevalent TB cases, (b) Incident TB cases, and (c) Controls. 7
- Table S4: Univariable and multivariable regression analyses of baseline predictors of RISK11 score in participants with prevalent TB at enrolment. 8
- Table S5: Univariable and multivariable regression analyses of baseline predictors of RISK11 score in participants who progressed to incident TB. 9
- Table S6: Univariable and multivariable regression analyses of baseline predictors of RISK11 score in participants without prevalent TB and did not progress to incident TB (controls). 10
- Table S7a: Initial exploratory ROC regression analysis for the effect of covariates on discriminatory accuracy of RISK11. 11
- Table S7b: Final ROC regression analysis for the effect of covariates on discriminatory accuracy of RISK11 derived from table 6a 12

Supplementary Figures 13

- Figure S1: Study design. 13
- Figure S2: Distribution of baseline RISK11 scores in (a) prevalent and (b) incident TB cases stratified by chest radiograph positivity at diagnosis. 14
- Figure S3: Distribution of baseline RISK11 scores among controls, one-sample sputum positive cases, and two-sample sputum positive cases unadjusted for sampling weights. 15

References 16

The CORTIS Study Team

First name	Surname	Affiliation
Craig	Innes	The Aurum Institute
William	Brumskine	The Aurum Institute
Kesenogile	Baepanye	The Aurum Institute
Tshepiso	Baepanye	The Aurum Institute
Ken	Clarke	The Aurum Institute
Marelize	Collignon	The Aurum Institute
Audrey	Dlamini	The Aurum Institute
Candice	Eyre	The Aurum Institute
Tebogo	Feni	The Aurum Institute
Moogo	Fikizolo	The Aurum Institute
Phinda	Galane	The Aurum Institute
Thelma	Goliath	The Aurum Institute
Alia	Gangat	The Aurum Institute
Shirley	Malefo-Grootboom	The Aurum Institute
Elba	Janse van Rensburg	The Aurum Institute
Bonita	Janse van Rensburg	The Aurum Institute
Sophy	Kekana	The Aurum Institute
Marietjie	Zietsman	The Aurum Institute
Adrianne	Kock	The Aurum Institute
Israel	Kunene	The Aurum Institute
Aneessa	Lakhi	The Aurum Institute
Nondumiso	Langa	The Aurum Institute
Hilda	Ledwaba	The Aurum Institute
Marillyn	Luphoko	The Aurum Institute
Immaculate	Mabasa	The Aurum Institute
Dorah	Mabe	The Aurum Institute
Nkosinathi	Mabuza	The Aurum Institute
Molly	Majola	The Aurum Institute
Mantai	Makhetha	The Aurum Institute
Mpho	Makoanyane	The Aurum Institute
Blossom	Makhubalo	The Aurum Institute
Vernon	Malay	The Aurum Institute
Juanita	Market	The Aurum Institute
Selvy	Matshego	The Aurum Institute
Nontsikelelo	Mbipa	The Aurum Institute
Tsiamo	Mmotsa	The Aurum Institute
Sylvester	Modipa	The Aurum Institute
Samuel	Mopati	The Aurum Institute
Palesa	Moswegu	The Aurum Institute
Primrose	Mothaga	The Aurum Institute
Dorothy	Muller	The Aurum Institute
Grace	Nchwe	The Aurum Institute
Maryna	Nel	The Aurum Institute
Lindiwe	Nhlangulela	The Aurum Institute
Bantubonke	Ntamo	The Aurum Institute
Lawerence	Ntoahae	The Aurum Institute
Tedrius	Ntshauba	The Aurum Institute
Nomsa	Sanyaka	The Aurum Institute
Letlhogonolo	Seabela	The Aurum Institute
Pearl	Selepe	The Aurum Institute
Melissa	Senne	The Aurum Institute
MG	Serake	The Aurum Institute
Maria	Thlapi	The Aurum Institute
Vincent	Tshikovhi	The Aurum Institute
Lebogang	Tswaile	The Aurum Institute
Amanda	van Aswegen	The Aurum Institute
Lungile	Mbata	The Aurum Institute
Constance	Takavamanya	The Aurum Institute
Pedro	Pinho	The Aurum Institute
John	Mdlulu	The Aurum Institute
Marthinette	Taljaard	The Aurum Institute
Naydene	Slabbert	The Aurum Institute
Sharfuddin	Sayed	The Aurum Institute
Tanya	Nielson	The Aurum Institute
Melissa	Senne	The Aurum Institute
Ni	Ni Sein	The Aurum Institute
Lungile	Mbata	The Aurum Institute
Dhineshree	Govender	Centre for the AIDS Programme of Research in South Africa (CAPRISA)

Tilagavathy	Chinappa	Centre for the AIDS Programme of Research in South Africa (CAPRISA)
Mbali Ignatia	Zulu	Centre for the AIDS Programme of Research in South Africa (CAPRISA)
Nonhle Bridgette	Maphanga	Centre for the AIDS Programme of Research in South Africa (CAPRISA)
Senzo Ralph	Hlathi	Centre for the AIDS Programme of Research in South Africa (CAPRISA)
Goodness Khanyisile	Gumede	Centre for the AIDS Programme of Research in South Africa (CAPRISA)
Thandiwe Yvonne	Shezi	Centre for the AIDS Programme of Research in South Africa (CAPRISA)
Jabulisiwe Lethabo	Maphanga	Centre for the AIDS Programme of Research in South Africa (CAPRISA)
Zandile Patrica	Jali	Centre for the AIDS Programme of Research in South Africa (CAPRISA)
Thobelani	Cwele	Centre for the AIDS Programme of Research in South Africa (CAPRISA)
Nonhlanhla Zanele Elsie	Gwamanda	Centre for the AIDS Programme of Research in South Africa (CAPRISA)
Celaphiwe	Dlamini	Centre for the AIDS Programme of Research in South Africa (CAPRISA)
Zibuyile Phindile Penlee	Sing	Centre for the AIDS Programme of Research in South Africa (CAPRISA)
Ntombozuko Gloria	Ntanjana	Centre for the AIDS Programme of Research in South Africa (CAPRISA)
Sphelele Simo	Nzimande	Centre for the AIDS Programme of Research in South Africa (CAPRISA)
Siyabonga	Mbatha	Centre for the AIDS Programme of Research in South Africa (CAPRISA)
Bhavna	Maharaj	Centre for the AIDS Programme of Research in South Africa (CAPRISA)
Atika	Moosa	Centre for the AIDS Programme of Research in South Africa (CAPRISA)
Cara-Mia	Corris	Centre for the AIDS Programme of Research in South Africa (CAPRISA)
Fazlin	Kafaar	South African Tuberculosis Vaccine Initiative, University of Cape Town
Marwou	De Kock	South African Tuberculosis Vaccine Initiative, University of Cape Town
Hennie	Geldenhuys	South African Tuberculosis Vaccine Initiative, University of Cape Town
Angelique Kany Kany	Luabeya	South African Tuberculosis Vaccine Initiative, University of Cape Town
Justin	Shenje	South African Tuberculosis Vaccine Initiative, University of Cape Town
Natasja	Botes	South African Tuberculosis Vaccine Initiative, University of Cape Town
Susan	Rossouw	South African Tuberculosis Vaccine Initiative, University of Cape Town
Hadn	Africa	South African Tuberculosis Vaccine Initiative, University of Cape Town
Bongani	Diamond	South African Tuberculosis Vaccine Initiative, University of Cape Town
Samentra	Braaf	South African Tuberculosis Vaccine Initiative, University of Cape Town
Sonia	Stryers	South African Tuberculosis Vaccine Initiative, University of Cape Town
Alida	Carstens	South African Tuberculosis Vaccine Initiative, University of Cape Town
Ruwiyda	Jansen	South African Tuberculosis Vaccine Initiative, University of Cape Town
Simbarashe	Mabwe	South African Tuberculosis Vaccine Initiative, University of Cape Town
Roxane	Herling	South African Tuberculosis Vaccine Initiative, University of Cape Town
Ashley	Veldsman	South African Tuberculosis Vaccine Initiative, University of Cape Town
Katie	Hadley	South African Tuberculosis Vaccine Initiative, University of Cape Town
Lebohgang	Makhete	South African Tuberculosis Vaccine Initiative, University of Cape Town
Chris	Hikuam	South African Tuberculosis Vaccine Initiative, University of Cape Town
Masooda	Kaskar	South African Tuberculosis Vaccine Initiative, University of Cape Town
Thelma	Leopeng	South African Tuberculosis Vaccine Initiative, University of Cape Town
Nicole	Bilek	South African Tuberculosis Vaccine Initiative, University of Cape Town
Mzwandile	Erasmus	South African Tuberculosis Vaccine Initiative, University of Cape Town
Lungisa	Jaxa	South African Tuberculosis Vaccine Initiative, University of Cape Town
Rodney	Raphela	South African Tuberculosis Vaccine Initiative, University of Cape Town
Fazlin	Kafaar	South African Tuberculosis Vaccine Initiative, University of Cape Town
Marcia	Steyn	South African Tuberculosis Vaccine Initiative, University of Cape Town
Sivuyile	Buhlungu	South African Tuberculosis Vaccine Initiative, University of Cape Town
Margareth	Erasmus	South African Tuberculosis Vaccine Initiative, University of Cape Town
Ilse	Davids	South African Tuberculosis Vaccine Initiative, University of Cape Town
Patiswa	Plaatjie	South African Tuberculosis Vaccine Initiative, University of Cape Town
Alessandro	Companie	South African Tuberculosis Vaccine Initiative, University of Cape Town
Frances	Ratangee	South African Tuberculosis Vaccine Initiative, University of Cape Town
Helen	Veldtsman	South African Tuberculosis Vaccine Initiative, University of Cape Town
Christel	Petersen	South African Tuberculosis Vaccine Initiative, University of Cape Town
Charmaine	Abrahams	South African Tuberculosis Vaccine Initiative, University of Cape Town
Miriam	Moses	South African Tuberculosis Vaccine Initiative, University of Cape Town
Xoliswa	Kelepu	South African Tuberculosis Vaccine Initiative, University of Cape Town
Yolande	Gregg	South African Tuberculosis Vaccine Initiative, University of Cape Town
Liticia	Swanepoel	South African Tuberculosis Vaccine Initiative, University of Cape Town
Nomsitho	Magawu	South African Tuberculosis Vaccine Initiative, University of Cape Town
Nompumelelo	Cetywayo	South African Tuberculosis Vaccine Initiative, University of Cape Town
Lauren	Mactavie	South African Tuberculosis Vaccine Initiative, University of Cape Town
Habibullah	Valley	South African Tuberculosis Vaccine Initiative, University of Cape Town
Elizabeth	Filander	South African Tuberculosis Vaccine Initiative, University of Cape Town
Nambitha	Nqakala	South African Tuberculosis Vaccine Initiative, University of Cape Town
Angelique	Mouton	South African Tuberculosis Vaccine Initiative, University of Cape Town
Fajwa	Opperman	South African Tuberculosis Vaccine Initiative, University of Cape Town
Elma	Van Rooyen	South African Tuberculosis Vaccine Initiative, University of Cape Town
Petrus	Tyambetyu	South African Tuberculosis Vaccine Initiative, University of Cape Town
Andriëtte	Hiemstra	DST/NRF Centre of Excellence for Biomedical TB Research and SAMRC Centre for TB Research, Stellenbosch University
Stephanus T	Malherbe	DST/NRF Centre of Excellence for Biomedical TB Research and SAMRC Centre for TB Research, Stellenbosch University

Razia	Hassan-Moosa	DST/NRF Centre of Excellence for Biomedical TB Research and SAMRC Centre for TB Research, Stellenbosch University
Elizna	Maasdorp	DST/NRF Centre of Excellence for Biomedical TB Research and SAMRC Centre for TB Research, Stellenbosch University
Justine	Khoury	DST/NRF Centre of Excellence for Biomedical TB Research and SAMRC Centre for TB Research, Stellenbosch University
Belinda	Kriel	DST/NRF Centre of Excellence for Biomedical TB Research and SAMRC Centre for TB Research, Stellenbosch University
Bronwyn	Smith	DST/NRF Centre of Excellence for Biomedical TB Research and SAMRC Centre for TB Research, Stellenbosch University
Liesel	Muller	DST/NRF Centre of Excellence for Biomedical TB Research and SAMRC Centre for TB Research, Stellenbosch University
Susanne	Tonsing	DST/NRF Centre of Excellence for Biomedical TB Research and SAMRC Centre for TB Research, Stellenbosch University
Andre	Loxton	DST/NRF Centre of Excellence for Biomedical TB Research and SAMRC Centre for TB Research, Stellenbosch University
Andriette	Hiemstra	DST/NRF Centre of Excellence for Biomedical TB Research and SAMRC Centre for TB Research, Stellenbosch University
Petri	Ahlers	DST/NRF Centre of Excellence for Biomedical TB Research and SAMRC Centre for TB Research, Stellenbosch University
Marika	Flinn	DST/NRF Centre of Excellence for Biomedical TB Research and SAMRC Centre for TB Research, Stellenbosch University
Eva	Chung	Vaccine and Infectious Disease Division, Fred Hutchinson Cancer Research Center
Michelle	Chung	Vaccine and Infectious Disease Division, Fred Hutchinson Cancer Research Center
Alicia	Sato	Vaccine and Infectious Disease Division, Fred Hutchinson Cancer Research Center
Steven	Self	Vaccine and Infectious Disease Division, Fred Hutchinson Cancer Research Center
Richard G.	White	TB Modelling Group, TB Centre, Centre for Mathematical Modelling of Infectious Diseases, Department of Infectious Disease Epidemiology, London School of Hygiene & Tropical Medicine

The CORTIS study team members[1]

Supplementary Tables

Table S1: The 11 transcripts comprising the RISK11 signature of TB risk and their putative functions.

Symbol	Description/ putative function(s)
BATF2	Homo sapiens basic leucine zipper transcription factor, ATF-like 2 (BATF2), mRNA; protein dimerization activity [goid 46983]v [evidence IEA]; sequence-specific DNA binding [goid 43565] [evidence IEA]; transcription factor activity [goid 3700] [evidence IEA]; regulation of transcription, DNA-dependent [goid 6355] [evidence IEA]; nucleus [goid 5634] [evidence IEA]
ETV7	Homo sapiens ets variant gene 7 (TEL2 oncogene) (ETV7), mRNA; sequence-specific DNA binding [goid 43565] [evidence IEA]; transcription factor activity [goid 3700] [evidence IEA]; specific RNA polymerase II transcription factor activity [goid 3704] [pmid 10828014] [evidence TAS]; transcription [goid 6350] [evidence IEA]; transcription from RNA polymerase II promoter [goid 6366] [pmid 10828014] [evidence TAS]; regulation of transcription, DNA-dependent [goid 6355] [evidence IEA]; nucleus [goid 5634] [pmid 10828014] [evidence TAS]
FCGR1C‡	Homo sapiens Fc Fragment Of IgG Receptor Ic, Pseudogene (FCGR1CP), mRNA; The gene represents one of three related immunoglobulin gamma Fc receptor genes located on chromosome 1. This family member lacks the transmembrane and coiled-coiled domains found in other family members and is thought to be a pseudogene of Fc-gamma-receptor 1A.
GBP1	Homo sapiens guanylate binding protein 1, interferon-inducible, 67kDa (GBP1), mRNA; GTP binding [goid 5525] [pmid 1715024] [evidence TAS]; GTPase activity [goid 3924] [evidence IEA]; nucleotide binding [goid 166] [evidence IEA]; immune response [goid 6955] [evidence IEA]; membrane [goid 16020] [evidence IEA]
GBP2	Homo sapiens guanylate binding protein 2, interferon-inducible (GBP2), mRNA; GTP binding [goid 5525] [pmid 1715024] [evidence TAS]; GTPase activity [goid 3924] [evidence IEA]; nucleotide binding [goid 166] [evidence IEA];immune response [goid 6955] [pmid 1715024] [evidence TAS]; membrane [goid 16020] [evidence IEA]
GBP5	Homo sapiens guanylate binding protein 5 (GBP5), mRNA; GTP binding [goid 5525] [evidence IEA]; GTPase activity [goid 3924] [evidence IEA]; nucleotide binding [goid 166] [evidence IEA]; immune response [goid 6955] [evidence IEA]; membrane [goid 16020] [evidence IEA]
SCARF1	Homo sapiens scavenger receptor class F, member 1 (SCARF1), transcript variant 3, mRNA; low-density lipoprotein binding [goid 30169] [pmid 9395444] [evidence IDA]; transmembrane receptor activity [goid 4888] [pmid 9395444] [evidence TAS]; protein binding [goid 5515] [evidence IEA]; low-density lipoprotein catabolism [goid 45192] [pmid 9395444] [evidence TAS]; cell adhesion [goid 7155] [evidence IEA]; receptor mediated endocytosis [goid 6898] [pmid 9395444] [evidence TAS]; membrane [goid 16020] [evidence IEA]; integral to membrane [goid 16021] [pmid 9395444] [evidenc
SERPING1	Homo sapiens serpin peptidase inhibitor, clade G (C1 inhibitor), member 1, (angioedema, hereditary) (SERPING1), transcript variant 2, mRNA; serine-type endopeptidase inhibitor activity [goid 4867] [pmid 1363816] [evidence TAS]; innate immune response [goid 45087] [evidence IEA]; blood coagulation [goid 7596] [evidence IEA]; circulation [goid 8015] [pmid 2563376] [evidence TAS]; complement activation, classical pathway [goid 6958] [evidence IEA]; extracellular region [goid 5576] [pmid 14718574] [evidence NAS]
STAT1	Homo sapiens signal transducer and activator of transcription 1, 91kDa (STAT1), transcript variant alpha, mRNA; transcription factor activity [goid 3700] [pmid 10848577] [evidence TAS]; hematopoietin/interferon-class (D200-domain) cytokine receptor signal transducer activity [goid 5062] [pmid 8608597] [evidence TAS]; signal transducer activity [goid 4871] [evidence IEA]; protein binding [goid 5515] [pmid 12867595] [evidence IPI]; calcium ion binding [goid 5509] [evidence IEA]; transcription from RNA polymerase II promoter [goid 6366] [pmid 9630226] [evidence TA
TAP1	Homo sapiens transporter 1, ATP-binding cassette, sub-family B (MDR/TAP) (TAP1), mRNA; ATPase activity, coupled to transmembrane movement of substances [goid 42626] [evidence IEA]; ATPase activity [goid 16887] [evidence IEA]; transporter activity [goid 5215] [evidence IEA]; nucleotide binding [goid 166] [evidence IEA]; protein heterodimerization activity [goid 46982] [pmid 11133832] [evidence IPI]; ATP binding [goid 5524] [evidence IEA]; oligopeptide transporter activity [goid 15198] [evidence IEA]; protein bin
TRAFD1	Homo sapiens TRAF-type zinc finger domain containing 1 (TRAFD1), mRNA; zinc ion binding [goid 8270] [evidence IEA]; nucleic acid binding [goid 3676] [evidence IEA]; intracellular [goid 5622] [evidence IEA]

Sources:

(a) Gene symbols and descriptions/function(s) are based on the Chaussabel et al gene set modules except for FCGR1C [2]
(b) FCGR1C‡ pseudogene: Gene symbol and description/function(s) are based on the GeneCards database (https://www.genecards.org/cgi-bin/carddisp.pl?gene=FCGR1CP) [3]. The most recent (third) iteration for the Chaussabel gene set modules (CM; 'BloodGen3') does not include FCGR1C [4,5]

Table S2: Baseline characteristics of enrolled participants by TB status adjusted to reflect screening population.

Variable	Total	a) Prevalent TB	b) Incipient TB	c) Control	a vs b vs c
	n=2923	n=74	n=56	n=2793	P-value
Age (median, IQR)	26 (22–33)	29 (24–36)	28 (22–37)	26 (22–33)	0·01
BMI (median, IQR)	23 (20–28)	21 (18–24)	20 (19–23)	23 (20–28)	0·01
RISK11 Score (median, IQR)	26 (8–77)	87 (61–96)	67 (15–81)	24 (8–75)	0·01
Sex (males) (n, %)	1338 (48·5)	47 (62·2)	33 (69·2)	1258 (48)	<0·001
Race (n, %)					
Caucasian	4 (0·2)	0 (0)	0 (0)	4 (0·2)	0·02
Mixed	968 (30·7)	34 (45·1)	26 (47·4)	908 (30·3)	
Black	1947 (69·0)	40 (54·9)	30 (52·6)	1877 (69·4)	
Asian	4 (0·2)	0 (0)	0 (0)	4 (0·2)	
Smoking history (n, %)	1478 (49·8)	45 (64·1)	41 (79·6)	1392 (49·2)	<0·001
Prior TB (n, %)	230 (7·0)	19 (20·6)	8 (19·4)	203 (6·6)	<0·001
TB contact history (n, %)	462 (16·0)	15 (21·4)	9 (11)	438 (16)	0·23
Flu-like symptoms (n, %)	134 (3·8)	4 (2·4)	1 (0·6)	129 (3·9)	0·87
TB Symptoms					
Chest pains (n, %)	30 (0·8)	4 (2·4)	0 (0)	26 (0·8)	0·20
Cough (n, %)	58 (1·6)	12 (7·1)	0 (0)	46 (1·5)	0·01
Fever (n, %)	3 (0·1)	1 (0·6)	0 (0)	2 (0·1)	1·00
Haemoptysis (n, %)	2 (0·1)	0 (0)	0 (0)	2 (0·1)	1·00
Loss of weight (n, %)	41 (1·3)	5 (3)	0 (0)	36 (1·3)	0·39
Night sweats (n, %)	32 (0·6)	7 (4·2)	1 (3·5)	24 (0·6)	0·01
Any symptom (n, %)	123 (3·4)	13 (7·7)	1 (3·5)	109 (3·4)	0·08

For continuous data, p values were computed using Wilcoxon Rank Sum test between two groups and Kruskal Wallis test for more than two groups. For categorical data, p values were computed using Fischer's exact test. IQR, inter-quartile range. BMI, body mass index.

Table S3: Comparison of distribution of RISK11 scores by baseline characteristics in (a) Prevalent TB cases, (b) Incident TB cases, and (c) Controls.

Variable	Category	(a) Prevalent TB, N = 74			(b) Incident TB, N = 56			(c) Controls, N = 2,793		
		n (%)	RISK11 score median (IQR)	P-value	n (%)	RISK11 score median (IQR)	P-value	n (%)	RISK11 score median (IQR)	P-value
Race	Caucasian	0	-	-	0	-	-	4 (0·1)	12·5 (5·6; 47·0)	ref
	Mixed	34 (46·0)	84·2 (61·4–94·8)		26 (46·6)	67·3 (33·3–80·1)		908 (32·5)	39·7 (12·1–76·2)	0·18
	Black	40 (54·0)	91·1 (58·9–97·0)	0·37	30 (53·4)	67·4 (8·2–90·0)	0·28	1,877(67·2)	18·2 (7·4–74·5)	0·42
	Asian	0	-	-	0	-	-	4 (0·1)	15·8 (8·8–60·6)	0·39
Sex	Female	27 (36·5)	90·9 (40·3–95·2)		23 (41·1)	69·7 (55·8–82·3)		1,535 (55·0)	34·1 (10·0–77·5)	
	Male	47 (63·5)	86·6 (61·4–96·9)	0·78	33 (58·9)	48·9 (7·8–80·1)	0·85	1,258 (45·0)	16·9 (6·9–71·4)	<0·001
Chest pains	No	70 (94·6)	85·7 (54·5–95·2)		56 (100)	67·3 (15·4–81·2)		2,767 (99·1)	24·2 (8·2–74·9)	
	Yes	4 (5·4)	98·3 (95·5–99·1)	0·02	0	-	-	26 (0·9)	28·4 (9·5–93·5)	0·22
Cough	No	62 (83·8)	83·5 (41·6–94·8)		56 (100)	67·3 (15·4–81·2)		2,747 (98·3)	23·8 (8·2–74·9)	
	Yes	12 (16·2)	96·1 (92·9–98·3)	0·001	0	-	-	46 (1·7)	41·3 (12·3–89·6)	0·08
Fever	No	73 (98·6)	86·6 (61·4–96·1)		56 (100)	67·3 (15·4–81·2)		2,791 (99·9)	24·2 (8·2–75·3)	
	Yes	1 (1·4)	92·6 (NA)	0·64	0	-	-	2 (0·1)	55·2 (24·7–85·7)	0·42
Flu-like symptoms	No	70 (94·6)	85·7 (54·5–95·7)		55 (98·2)	67·1 (15·2–80·1)		2,664 (95·4)	22·9 (8·2–74·2)	
	Yes	4 (5·4)	96·3 (93·5–98·9)	0·05	1 (1·8)	99·6 (NA)	0·13	129 (4·6)	61·0 (12·1–88·3)	<0·001
Haemoptysis	No	74 (100)	87·0 (61·4–96·1)		56 (100)	67·3 (15·4–81·2)		2,791 (99·9)	24·2 (8·2–75·3)	
	Yes	0	-	-	0	-	-	2 (0·1)	7·4 (6·9–7·8)	0·15
Loss of weight	No	69 (93·2)	85·7 (54·5–95·2)		56 (100)	67·3 (15·4–81·2)		2,757 (98·7)	24·2 (8·2–74·9)	
	Yes	5 (6·8)	97·0 (94·4–98·3)	0·02	0	-	-	36 (1·3)	19·3 (10·0–82·0)	0·66
Night sweats	No	67 (90·5)	85·7 (51·1–94·8)		55 (98·2)	67·5 (15·2–82·3)		2,769 (99·1)	23·7 (8·2–74·9)	
	Yes	7 (9·5)	98·3 (92·6–100)	0·01	1 (1·8)	45 (NA)	0·64	24 (0·9)	71·4 (28·6–94·6)	0·003
Smoking history	No	29 (39·2)	92·6 (68·4–97·8)		15 (26·8)	68·8 (55·8–90·0)		1,401 (50·2)	22·0 (8·2–74·5)	
	Yes	45 (60·8)	82·7 (51·1–93·5)	0·13	41 (73·2)	66·1 (8·2–80·1)	0·37	1,392 (49·8)	26·4 (8·2–75·8)	0·31
Prior TB	No	55 (74·3)	89·5 (51·1–97·4)		48 (85·7)	70·1 (14·9–86·1)		2,590 (92·7)	22·7 (8·2–74·9)	
	Yes	19 (25·7)	85·7 (65·8–90·9)	0·29	8 (14·3)	42·2 (15·4–65·2)	0·27	203 (7·3)	42·9 (11·7–77·7)	0·02
TB contact history	No	59 (79·7)	85·7 (61·4–95·7)		47 (83·9)	67·5 (15·2–90·0)		2,355 (84·3)	25·1 (8·2–75·3)	
	Yes	15 (20·3)	91·3 (40·3–100·0)	0·28	9 (16·1)	66·1 (61·9–79·6)	0·92	438 (15·7)	21·2 (8·2–73·6)	0·72
Any symptom	No	61 (82·4)	81·8 (41·6–93·5)		55 (98·2)	67·5 (15·2–82·3)		2,684 (96·1)	23·4 (8·2–74·5)	
	Yes	13 (17·6)	97·0 (93·1–98·3)	<0·001	1 (1·8)	45 (NA)	0·64	109 (3·9)	48·5 (10·8–89·2)	0·002

P values were computed using Wilcoxon Rank Sum test for continuous data and Fischer's exact for categorical data. Data are not adjusted to reflect screening population.

Table S4: Univariable and multivariable regression analyses of baseline predictors of RISK11 score in participants with prevalent TB at enrolment.

Variable	n=74	Univariable Analysis		Multivariable Analysis		
		β. Coef. (95% CI)	P-value	β. Coef. (95% CI)	% marginal effect (95% CI)	P-value
Age (median, IQR)	29 (24–36)	-0·03 (-0·07–0·01)	0·04	-0·03 (-0·06–0·01)	-0·65 (-1·34–0·04)	0·07
BMI (median, IQR)	20·7 (18·5–23·8)	-0·07 (-0·16–0·01)	0·08	-	-	-
Male sex (n, %)	47 (63·5)	0·24 (-0·52–1)	0·53	-	-	-
Race:						
Black (n, %)	40 (54)	Ref	-	-	-	-
Mixed (n, %)	34 (46)	0·1 (-0·62–0·83)	0·78	-	-	-
Smoking history (n, %)	45 (60·8)	-0·24 (-1·05–0·56)	0·56	-	-	-
Prior TB (n, %)	19 (25·7)	-0·12 (-1·15–0·91)	0·82	-	-	-
TB contact history (n, %)	15 (20·7)	0·08 (-0·77–0·93)	0·85	-	-	-
Flu-like symptoms (n, %)	4 (5·4)	3·46 (2·62–4·31)	<0·001	-	-	-
Chest pains (n, %)	4 (5·4)	3·81 (2·71–4·91)	<0·001	-	-	-
Cough (n, %)	12 (16·2)	3·31 (2·64–3·98)	<0·001	3·23 (2·51–3·94)	72·55 (58·06–87·03)	<0·001
Fever (n, %)	1 (1·4)	2·72 (2·36–3·09)	<0·001	-	-	-
Loss of weight (n, %)	5 (6·8)	3·54 (2·77–4·32)	<0·001	-	-	-
Night sweats (n, %)	7 (9·5)	3·33 (2·26–4·4)	<0·001	-	-	-

IQR, inter-quartile range. BMI, body-mass index. β. Coef., Beta coefficient. % marginal effect, predicted marginal change in RISK11 score associated with each predictor variable in the model. P values are reported from the model output.

Table S5: Univariable and multivariable regression analyses of baseline predictors of RISK11 score in participants who progressed to incident TB.

Variable	n=56	Univariable Analysis		Multivariable Analysis		
		β. Coef. (95% CI)	P-value	β. Coef. (95% CI)	% marginal effect (95% CI)	P-value
Age (median, IQR)	28 (22-37)	0·02 (0–0·05)	0·10	-	-	-
BMI (median, IQR)	19·8 (18·7-23·4)	0·02 (-0·07–0·12)	0·61	-	-	-
Race:						
Male sex (n, %)	33 (58·9)	-0·75 (-1·51–0·01)	0·05	-	-	-
Race:						
Black (n, %)	30 (53·4)	Ref	-	-	-	-
Mixed (n, %)	26 (46·6)	0·5 (-0·2–1·19)	0·16	-	-	-
Smoking history (n, %)	41 (73·2)	-0·67 (-1·56–0·23)	0·14	-	-	-
Prior TB (n, %)	8 (14·3)	-0·03 (-0·87–0·8)	0·94	-	-	-
TB contact history (n, %)	9 (16·1)	0·03 (-1·25–1·31)	0·97	-	-	-
Flu-like symptoms (n, %)	1 (1·8)	6·33 (5·97–6·69)	<0·001	-	-	-
Night sweats (n, %)	1 (1·8)	0·7 (0·33–1·06)	<0·001	-	-	-

IQR, inter-quartile range. BMI, body-mass index. β. Coef., Beta coefficient. % marginal effect, predicted marginal change in RISK11 score associated with each predictor variable in the model. P values are reported from the model output.

Table S6: Univariable and multivariable regression analyses of baseline predictors of RISK11 score in participants without prevalent TB and did not progress to incident TB (controls).

Variable		n=2,793	Univariable Analysis		Multivariable Analysis		
			β. Coef. (95% CI)	P-value	β. Coef. (95% CI)	% marginal effect (95% CI)	P-value
Age (median, IQR)		26 (22-33)	0·01 (-0·01–0·01)	0·07	-		-
BMI (median, IQR)		22·7 (20-27·9)	0·01 (-0·01–0·01)	0·88	-		-
Male sex (n, %)		1258 (45)	-0·3 (-0·38–-0·21)	<0·001	-0·36 (-0·45–-0·27)	-5·99 (-7·49–-4·5)	<0·001
Race:	Black (n, %;)	1877 (67·2)	Ref	-	-		-
	Asian (n, %)	4 (0·1)	-0·14 (-0·94–0·66)	0·73	-		-
	Caucasian (n, %)	4 (0·1)	-0·47 (-1·32–0·37)	0·27	-		-
	Mixed (n, %)	908 (32·5)	0·36 (0·27–0·45)	<0·001	-		-
Smoking history (n, %)		1392 (49·8)	0·05 (-0·03–0·14)	0·20	0·16 (0·07–0·25)	2·74 (1·24–4·24)	<0·001
Prior TB (n, %)		20 (7·3)	0·22 (0·05–0·38)	0·01	0·18 (0·01–0·35)	3·03 (0·25–5·82)	0·03
TB contact history (n, %)		438 (15·7)	-0·07 (-0·18–0·04)	0·23	-		-
Flu-like symptoms (n, %)		129 (4·2)	0·31 (0·1–0·52)	<0·001	0·26 (0·05–0·48)	4·39 (0·08–7·98)	0·02
Chest pains (n, %)		26 (0·9)	0·03 (-0·4–0·46)	0·89	-		-
Cough (n, %)		46 (1·7)	0·26 (-0·06–0·59)	0·11	-		-
Fever (n, %)		2 (0·1)	0·60 (-0·31–1·51)	0·19	-		-
Haemoptysis (n, %)		2 (0·1)	-1·23 (-1·33–-1·13)	<0·001	-		-
Loss of weight (n, %)		36 (1·3)	-0·01 (-0·36–0·34)	0·95	-		-
Night sweats (n, %)		24 (0·9)	0·68 (0·08–1·29)	0·03	0·76 (0·14–1·38)	12·69 (2·34–23·04)	0·02

IQR, inter-quartile range. BMI, body-mass index. β. Coef., Beta coefficient. % marginal effect, predicted marginal change in RISK11 score associated with each predictor variable in the model. P values are reported from the model output.

Table S7a: Initial exploratory ROC regression analysis for the effect of covariates on discriminatory accuracy of RISK11.

Group/Variable	Prevalent TB		Incident TB	
	β. Coef. (95% CI)	P-value	β. Coef. (95% CI)	P-value
**Case population**				
BMI	-1·69 (-3·60–0·22)	0·08	-0·65 (-2·78–1·48)	0·55
Male sex	9·32 (-7·31–25·95)	0·27	-8·57 (-29·85–12·7)	0·43
Night sweats	-6·00 (-27·70–15·71)	0·59	10·6 (-6·61–27·81)	0·23
Flu-like symptoms	19·19 (-16·9–55·28)	0·30	60·14 (41·22–79·06)	<0·001
Haemoptysis	Omitted	-	Omitted	-
Smoking history	-9·39 (-24·01–5·23)	0·21	-13·24 (-37·84–11·36)	0·29
Cough	35·31 (14·78–55·83)	0·001	Omitted	-
**Control population**				
BMI	-0·11 (-0·21–-0·01)	0·04	-0·09 (-0·19–0·01)	0·06
Male sex	-6·70 (-8·30–-5·09)	<0·001	-6·54 (-8·13–-4·94)	<0·001
Night sweats	12·92 (-1·38–27·22)	0·08	11·51 (-3·79–26·8)	0·14
Flu-like symptoms	5·11 (0·98–9·23)	0·02	5·26 (1·09–9·43)	0·01
Haemoptysis	-12·82 (-20·07–-5·57)	0·001	-13·24 (-20·62–-5·86)	<0·001
Smoking history	2·67 (1·16–4·18)	0·001	2·71 (1·2–4·22)	<0·001
Cough	3·63 (-3·6–10·86)	0·33	4·23 (-3·14–11·6)	0·26
**ROC model**				
BMI	-0·06 (-0·12–0·01)	0·09	-0·02 (-0·1–0·05)	0·55
Male sex	0·32 (-0·26–0·89)	0·28	-0·31 (-1·11–0·48)	0·44
Night sweats	-0·2 (-0·94–0·53)	0·59	0·39 (-0·26–1·04)	0·24
Flu-like symptoms	0·65 (-0·57–1·87)	0·28	2·21 (1·4–3·01)	<0·001
Haemoptysis	Omitted	-	Omitted	-
Smoking history	-0·32 (-0·82–0·18)	0·21	-0·49 (-1·4–0·43)	0·30
Cough	1·19 (0·76–1·93)	0·001	Omitted	-

Covariates under 'case population' are those that affect TB cases and variables under 'control population' are those that affect controls without TB. The covariates under the 'ROC model' are those that affect discrimination between cases and controls. See online supplementary table 6b for the final ROC regression model derived from this analysis by removing the non-significant variables in each category. P values are reported from the model output.
BMI, body mass index.

Table S7b: Final ROC regression analysis for the effect of covariates on discriminatory accuracy of RISK11 derived from table 7a

Group/Variable	Prevalent TB		Incident TB	
	β. Coef. (95% CI)	P-value	β. Coef. (95% CI)	P-value
**Case population**				
Cough	43·5 (30·64–56·37)	<0·001	-	-
Flu-like symptoms	-	-	50·38 (40·26–60·49)	<0·001
**Control population**				
BMI	-0·11 (-0·21–0)	0·04	-0·15 (-0·27–-0·03)	0·02
Male sex	-6·57 (-8·17–-4·96)	<0·001	-7·72 (-9·76–-5·68)	<0·001
Night Sweats	15·02 (2·12–27·91)	0·02	17·71 (2·71–32·7)	0·02
Flu-like symptoms	5·17 (1·05–9·29)	0·014	8·4 (2·89–13·91)	0·003
Haemoptysis	-9·31 (-10·67–-7·96)	<0·001	-12·88 (-14·67–-11·1)	<0·001
Smoking history	2·64 (1·14–4·14)	0·001	3·15 (1·21–5·08)	0·001
**ROC model**				
Cough	1·40 (1·00–1·87)	<0·001	-	-
Flu-like symptoms	-	-	1·58 (1·14–2·03)	<0·001

Covariates under 'case population' are those that affect TB cases and variables under 'control population' affect controls without TB. The covariates under the 'ROC model' are those that affect discrimination between cases and controls. See online supplementary table 6a for detailed model on which this model is based. P values are reported from the model output.

BMI, body-mass Index.

Supplementary Figures

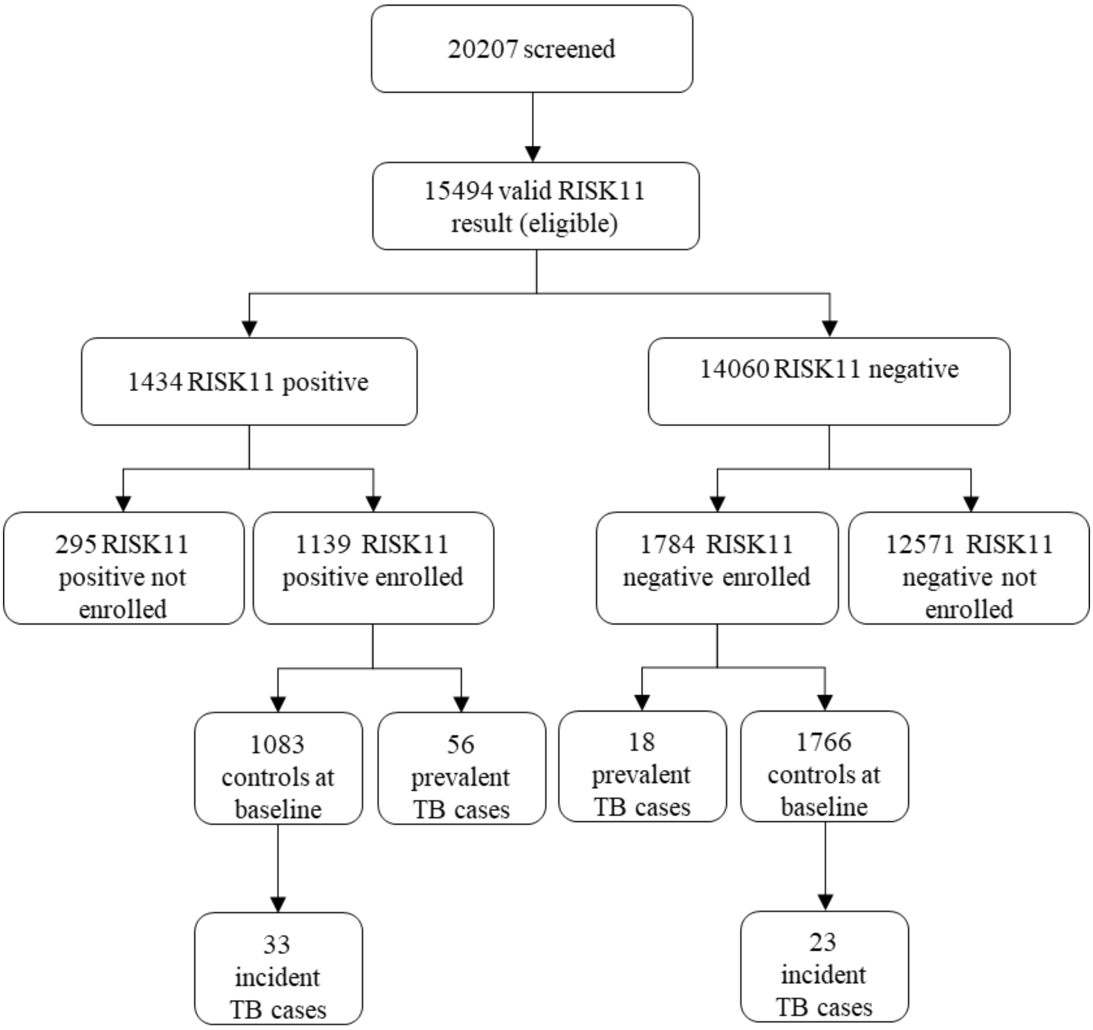

Figure S1: Study design.

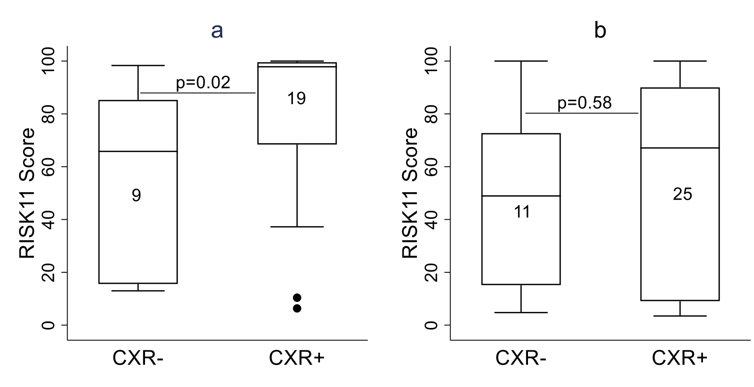

Figure S2: Distribution of baseline RISK11 scores in (a) prevalent and (b) incident TB cases stratified by chest radiograph positivity at diagnosis.

CXR+, chest radiograph suggestive of TB disease. CXR-, chest radiograph not suggestive of TB disease. P values were computed using Wilcoxon Rank Sum.

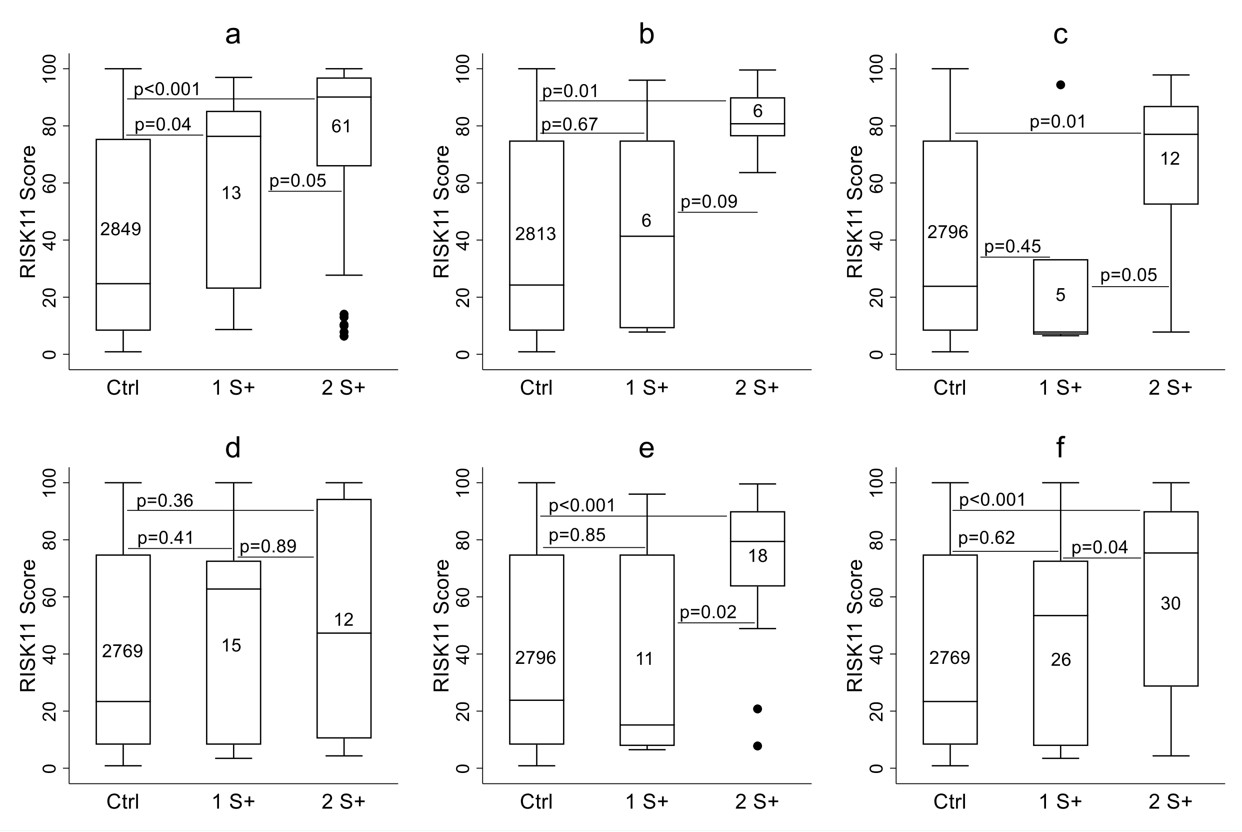

Figure S3: Distribution of baseline RISK11 scores among controls, one-sample sputum positive cases, and two-sample sputum positive cases unadjusted for sampling weights.

a) RISK11 score distribution in controls and prevalent TB cases (TB cases at baseline). b) RISK11 score distribution in controls and incident TB cases diagnosed between months two and six. c) RISK11 score distribution in controls and incident TB cases diagnosed between months seven and twelve. d) RISK11 score distribution in controls and incident TB cases diagnosed between months thirteen and fifteen. e) RISK11 score distribution in controls and cumulative incident TB cases diagnosed between months one and twelve. f) RISK11 score distribution in controls and cumulative incident TB cases diagnosed between months one and fifteen. Numbers in b, c, d, e and f excludes 24 participants who did not attend a subsequent visit and hence have unknown TB outcome. Numbers in the boxplots indicate the number of participants in that strata. P values were computed using Wilcoxon Rank Sum.

Ctrl, controls without TB. 1 S+, one-sample sputum positive TB cases. 2 S+, two-sample sputum positive TB cases.

References

[1] Scriba TJ, Fiore-Gartland A, Penn-Nicholson A, et al. Biomarker-guided tuberculosis preventive therapy (CORTIS): a randomised controlled trial. The Lancet Infectious diseases. 2021.

[2] Chaussabel D, Quinn C, Shen J, et al. A modular analysis framework for blood genomics studies: application to systemic lupus erythematosus. Immunity. 2008;29(1):150-64.

[3] Stelzer G, Rosen N, Plaschkes I, et al. The GeneCards Suite: From Gene Data Mining to Disease Genome Sequence Analyses. Curr Protoc Bioinformatics. 2016;54:1 30 1-1 3.

[4] Rinchai D, Roelands J, Toufiq M, et al. BloodGen3Module: blood transcriptional module repertoire analysis and visualization using R. Bioinformatics (Oxford, England). 2021;37(16):2382-9.

[5] Altman MC, Rinchai D, Baldwin N, et al. Development of a fixed module repertoire for the analysis and interpretation of blood transcriptome data. Nature communications. 2021;12(1):4385.
